# Status of Marine Biodiversity of the China Seas

**DOI:** 10.1371/journal.pone.0050719

**Published:** 2013-01-08

**Authors:** J. Y. Liu

**Affiliations:** Institute of Oceanology, Chinese Academy of Sciences, Qingdao, China; Université du Québec à Rimouski, Canada

## Abstract

China's seas cover nearly 5 million square kilometers extending from the tropical to the temperate climate zones and bordering on 32,000 km of coastline, including islands. Comprehensive systematic study of the marine biodiversity within this region began in the early 1950s with the establishment of the Qingdao Marine Biological Laboratory of the Chinese Academy of Sciences. Since that time scientists have carried out intensive multidisciplinary research on marine life in the China seas and have recorded 22,629 species belonging to 46 phyla. The marine flora and fauna of the China seas are characterized by high biodiversity, including tropical and subtropical elements of the Indo-West Pacific warm-water fauna in the South and East China seas, and temperate elements of North Pacific temperate fauna mainly in the Yellow Sea. The southern South China Sea fauna is characterized by typical tropical elements paralleled with the Philippine-New Guinea-Indonesia Coral triangle typical tropical faunal center.

This paper summarizes advances in studies of marine biodiversity in China's seas and discusses current research mainly on characteristics and changes in marine biodiversity, including the monitoring, assessment, and conservation of endangered species and particularly the strengthening of effective management. Studies of (1) a tidal flat in a semi-enclosed embayment, (2) the impact of global climate change on a cold-water ecosystem, (3) coral reefs of Hainan Island and Xisha-Nansha atolls, (4) mangrove forests of the South China Sea, (5) a threatened seagrass field, and (6) an example of stock enhancement practices of the Chinese shrimp fishery are briefly introduced. Besides the overexploitation of living resources (more than 12.4 million tons yielded in 2007), the major threat to the biodiversity of the China seas is environmental deterioration (pollution, coastal construction), particularly in the brackish waters of estuarine environments, which are characterized by high productivity and represent spawning and nursery areas for several economically important species. In the long term, climate change is also a major threat. Finally, challenges in marine biodiversity studies are briefly discussed along with suggestions to strengthen the field. Since 2004, China has participated in the Census of Marine Life, through which advances in the study of zooplankton and zoobenthos biodiversity were finally summarized.

## Introduction

China occupies the eastern part of the Eurasian continent adjacent to the western Pacific Ocean, including its marginal seas—the Bohai Sea, Yellow Sea, East China Sea, and South China Sea. The Chinese coastline of 32,000 km includes the coastlines of 6,500 islands. The coastline of mainland China runs 18,000 km from the mouth of the Yalu River on the China-Korea border in the north, to the mouth of the Beilun River on the China-Vietnam border in the south. The China seas cover an area of 4.73 million square kilometers across three climate zones—the temperate, subtropical, and tropical—from 3° to 41°N latitude [1].

The coastline features are affected by monsoon winds and Pacific tidal waves and currents and by several large rivers, including the Huanghe (Yellow River), Changjiang (Yangtze River), and Zhujiang (Pearl River). A corresponding variety of coastal ecosystems includes the vast area of flat coasts with either sandy beaches and barriers or wide tidal flats in northern China, indented coasts in the mountainous and hilly areas mainly in southern China, deltas and estuaries, coral reefs and mangroves, as well as seagrass fields and algal beds.

The coastline of China can be classified into two major types: the bedrock-embayed coast and the plains coast. The distribution of these types is controlled by geological and tectonic features, particularly zones of uplift and subsidence. The sedimentary processes of the coast of China are greatly affected by the large rivers (e.g., the Pearl, Yangtze, Yellow, Haihe, Liaohe, Yalu, and Heilong) that deliver enormous amounts of sediment. This sediment is redistributed by monsoon waves and tidal currents. Within tropical and subtropical climate zones, the growth of mangroves and corals is also an important factor in coastline formation—the biogenic coast [1], [2].

### Geological features of the China seas

The bottom geomorphology of the China seas becomes deeper from northwest to southeast. The continental shelf of the China seas is one of the widest in the world, embracing all of the Bohai and Yellow seas, two-thirds of the East China Sea, and more than half of the South China Sea. The shelf on the east coast of Taiwan is narrow, just over 10 km at the widest point. Below the continental shelf, the continental slope of the East China Sea off the east coast of Taiwan merges with the abrupt and narrow steps of the slope and the trough in the South China Sea. The continental slope of the South China Sea ranges in depth from 800 to 4,200 m, with a maximum slope of 4 degrees. Another feature of the China seas is the troughs. The Okinawa Trough in the East China Sea is 2,719 m deep while in the South China Sea, the Xisha, Zhongsha, Nansha, and Liyue West troughs are for the most part canyons produced by the expansion of that sea. The continental margin basin in the middle of the South China Sea is about 1,500 km long, widest in the north (820 km) with a plain bottom. The depth of the basin varies from 3,400–4,000 m in the north to 4,300–4,400 m in the south, with a series of seamounts running east to west and northeast to northwest. In the eastern part of the basin, a seamount consisting of tholeiite emerges from the sea surface to form Huangyandao Island [1].

### Current systems

**Figure 1:**
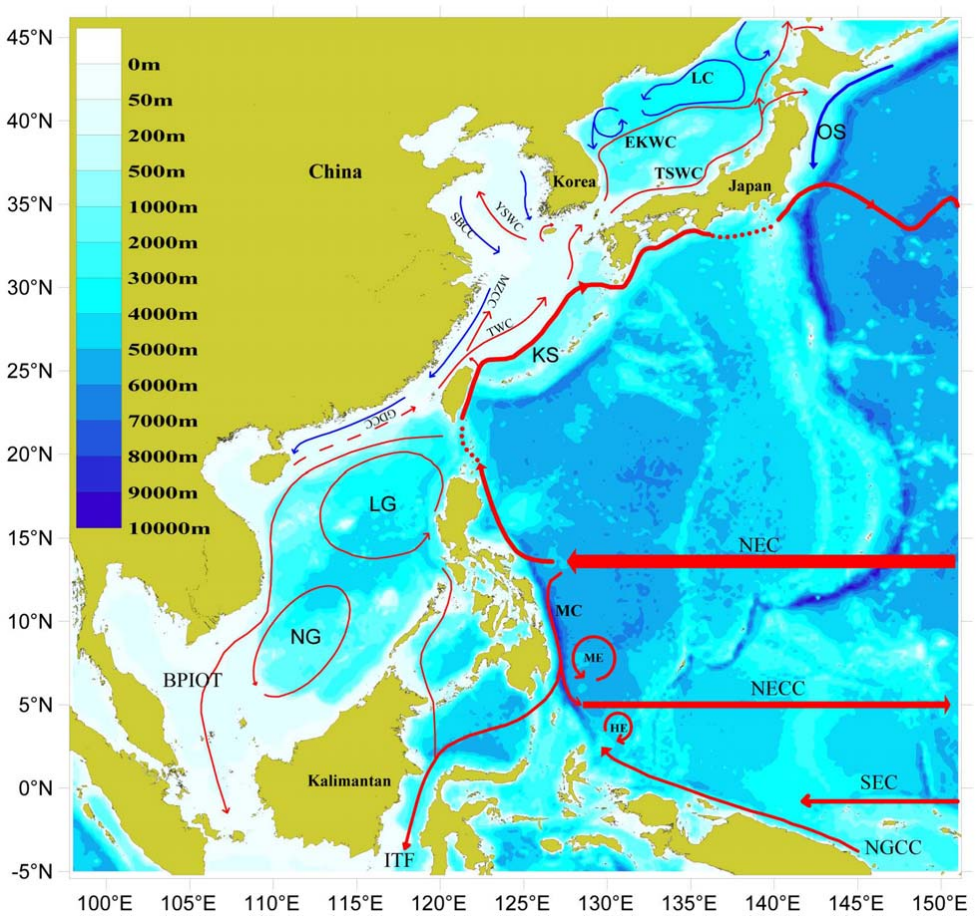
Schematic presentation of the major surface currents in the western Pacific Ocean in winter. (drawn by Yang Dezhou).

The two main current systems in the China seas are the Kuroshio and the coastal currents, both of which are characterized by cyclonic circulation [3]–[5] (Figure 1). The Kuroshio includes a strong main stream and its branches—the Taiwan Warm Current, the Tsushima Warm Current, and the Yellow Sea Warm Current. The Kuroshio is the continuation and northward branch of the North Equatorial Current. It flows northward along the east coast of Taiwan, the continental slope, and the outer shelf and then turns northeast to the southern coast of Kyushu, Japan. The maximum speed of the Kuroshio off Luzon Island is 100 cm sec^−1^ and may be as high as 150 cm sec^−1^ in the Luzon Strait and east off Taiwan. The speed decreases to 50–100 cm sec^−1^ while entering the East China Sea, then increases again near 26°39′N and 126°E. In the East China Sea, the main stream is 70–110 km wide with a speed greater than 40 cm sec^−1^. The water temperature is about 20°C at a depth of 100 m and 18°C at 200 m. Generally, there is no regularity to seasonal changes in the main stream. In certain years, the current is stronger in winter than in summer, but in other years, it may be stronger in summer and autumn or it may not change from summer to winter. However, the branch currents exhibit distinct seasonal variation. The maximum speed of the Tsushima Current is observed in September and the minimum in February. The Yellow Sea Current and the Taiwan Current are also generally stronger in winter than in summer. On these currents, a great number of warm-water marine species are transported from their tropical center to the north and expand their distribution ranges.

The coastal current systems consist of the Yellow Sea coastal current and the East China Sea and South China Sea coastal currents. The first of these is a brackish water current of low salinity (also low temperature in winter) flowing along the coasts of Shandong and Jiangsu provinces. It issues from Bohai Bay, flowing seasonally along the north coast of Shandong Peninsula. After turning south and westward from the Chengshan Cape and flowing along the Shandong coast, it turns south from the Haizhou Bay and flows to the north of the Yangtze River estuary and then turns southwest. From there, one part of the Yellow Sea coastal current joins the Yellow Sea Warm Current, and the other part joins the East China Sea coastal current at a speed of less than 25 cm sec^−1^. The East China Sea coastal current is a mixture of freshwater streams from the Qiantang and Minjiang rivers with low-salinity waters. The water in this current is characterized by high turbidity, large annual swings in temperature, and a speed of about 25 cm sec^−1^. The direction of the current changes seasonally from southward with the wind direction in winter to northward or to northeastward in summer. The coastal current transports temperate cold-water drifting species to the northern coast of the South China Sea.

The South China Sea coastal current flows from the area west of 116°E along Guangdong Province. It is characterized by low salinity (only 12 ppt in summer) and high speed, which averages 25 cm sec^−1^ near the Pearl River estuary. The highest speed is 70 cm sec^−1^. The current changes direction seasonally, flowing southwestward to Zhanjiang in winter and southward along Leizhou Peninsula before dividing into two branches. One branch flows southwest along the Hainan coast while the other turns northeast, forming Zhanjiang gyre, and flows to the northeast in summer.

The seasonal variation of coastal currents is influenced by the continental streams and also by monsoons. From November to February, coastal currents are affected by the strong north winter monsoon. The north-south wind is strongest and broadest. In the transfer period from March to May, the southward-flowing coastal current weakens and shrinks in Hangzhou Bay. During the southern monsoon from June to August, the southward-flowing coastal current weakens. South of Hangzhou Bay, the coastal currents of the East and South China seas mix with the offshore warm current system and flow northward. In September and October, the direction of monsoon changes from north to the south, and the southward flow of winter coastal current strengthens and expands [4] [6].

### The Bohai Sea: China's Inland Sea

The Bohai Sea is the northernmost part of the China seas (37°07′–41°0′N, 117°35′–121°10′E) and covers an area of 78,000 km^2^. It is enclosed by the North China Plain and is bordered by Liaoning, Hebei, and Shandong provinces and the city of Tianjin. It lies between the Liaodong and Shandong peninsulas and is connected with the Yellow Sea through the Bohai Strait between the Laotieshan Cape of Lushun Harbor in the north and the Penglai Cape in the south. The strait has a width of 59 nautical miles and includes about 30 scattered islets—the Miaodao Islands (with eight major islands). About 95 percent of the total area is a shallow sea of less than 30 m depth and an average depth of 18 m. The area of deepest water (86 m) is in the Laotieshan Channel [1].

Physical features of the Bohai Sea may be divided into five natural parts: the Bohai Bay in the northwest, the Liaodong Bay in the north, the Laizhou Bay in the south, the central basin, and the Bohai Strait in the east. The seafloor slopes gently toward the strait at 0°0′28″. Six channels for water exchange are found between the main islands along the strait. The northernmost of them, the Laotieshan Channel, is the main route by which the Yellow Sea Warm Current enters the Bohai Sea.

**Figure 2:**
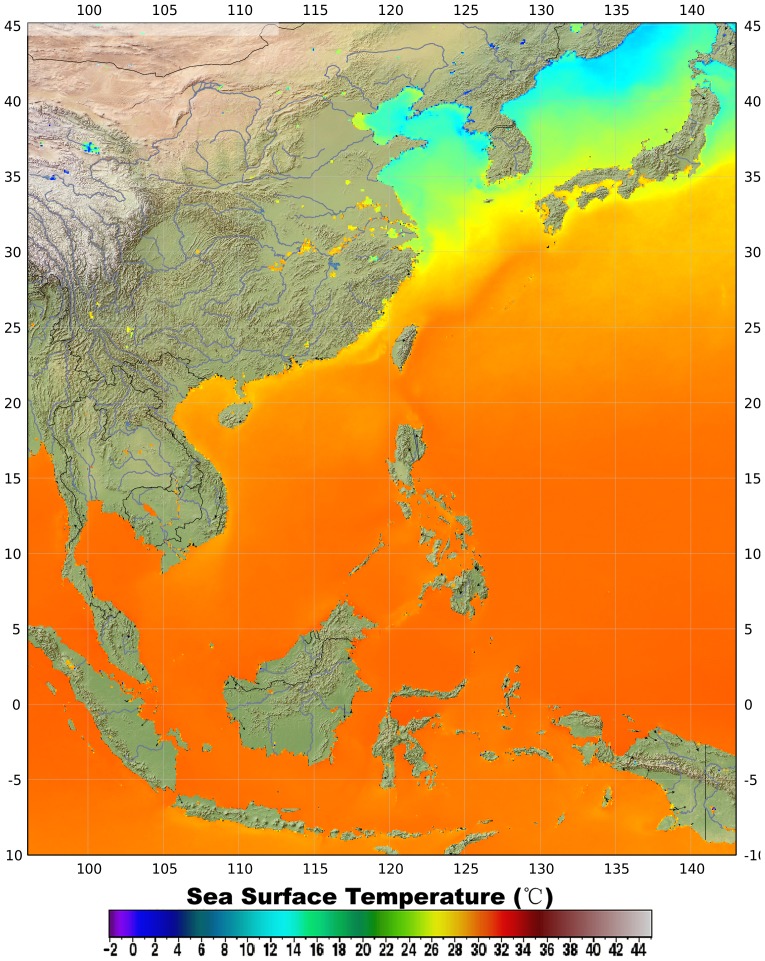
Surface water temperature in western Pacific Ocean in winter (2002–2011), derived from MODIS-Aqua satellite.

Hydrographic characteristics of the Bohai Sea are influenced mainly by the continental weather. The average water temperature is only 11°C, ranging from 0 to 21 degrees (Figure 2). Water temperatures are lowest in February and highest in August. Except for Qinhuangdao and Huludao harbors, a wide area of marginal ice is found in winter in the coastal zone. The predominantly low salinity of Bohai Sea water results from the freshwater discharged by many rivers, including the Huanghe (Yellow River), Haihe, Lanhe and Liaohe rivers. Eurythermal elements of the warm-water and temperate fauna and flora and low-salinity species dominate the area [7], [8].

### The Yellow Sea

The Yellow Sea is a semi-enclosed shallow sea situated between the northern part of mainland China and the Korea Peninsula (31°40′–39°50′N, 119°35′–126°50′E). The sea is bordered on the west by the North Jiangsu Plain and Shandong Peninsula, on the east by the Korean Peninsula, and on the north by Liaodong Peninsula. The sea covers an area of about 380,000 km^2^, the deepest part of which is in the southeast with a depth of about 140 m. The sea may be divided into north and south parts by a line from the Chengshan Cape, China, to the Changshanchuan Islands, Korea. The North Yellow Sea is semi-enclosed by the Shandong and Liaodong peninsulas in the west and north and by the Korean Peninsula in the east. It covers an area of about 80,000 km^2^ at an average depth of 38 m. The deepest area of the North Yellow Sea is in the southwest, off Bailingdao Island. The South Yellow Sea borders the East China Sea along a line from the mouth of the Yangtze River to Cheju Island. The South Yellow Sea covers about 300,000 km^2^ at an average depth of 45.3 m. It is deepest in the Yellow Sea Trough, with a maximum depth of 140 m in the north, off Cheju Island. The bottom slopes gently downward to the southeast toward the central area.

The Yellow Sea connects with the East China Sea on the south and with the Sea of Japan through the Korea Strait in the southeast. The bottom topography is open and slopes gently from the continent toward the southeast at 0°01′21″. The bottom sediment is silt and ooze from continental rivers [1], [9].

Hydrographic characteristics of the Yellow Sea are influenced mainly by the continent, particularly in the western part, where the temperature, salinity, and current show distinct seasonal changes. Annual variation of water temperature in the Yellow Sea is less than that in the Bohai Sea and ranges between 1–4°C in winter (Figure 2) and 24–28°C in summer. The salinity averages 32 ppt.

**Figure 3:**
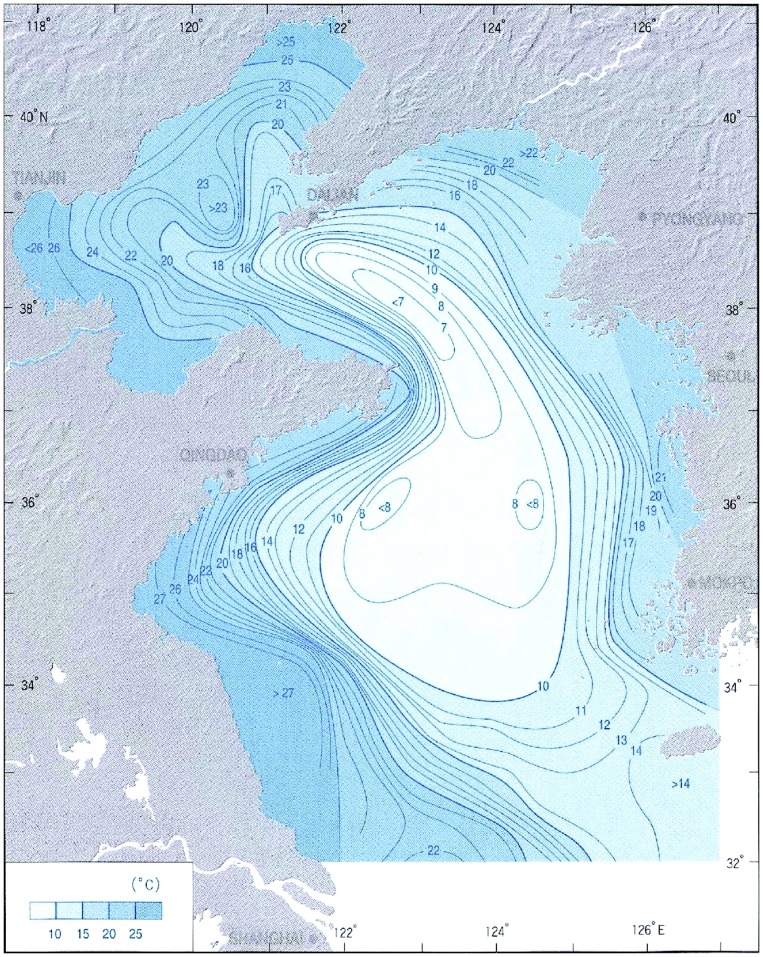
Bottom water temperature of Yellow Sea (August) (1958–1988) [**10**].

**Figure 4:**
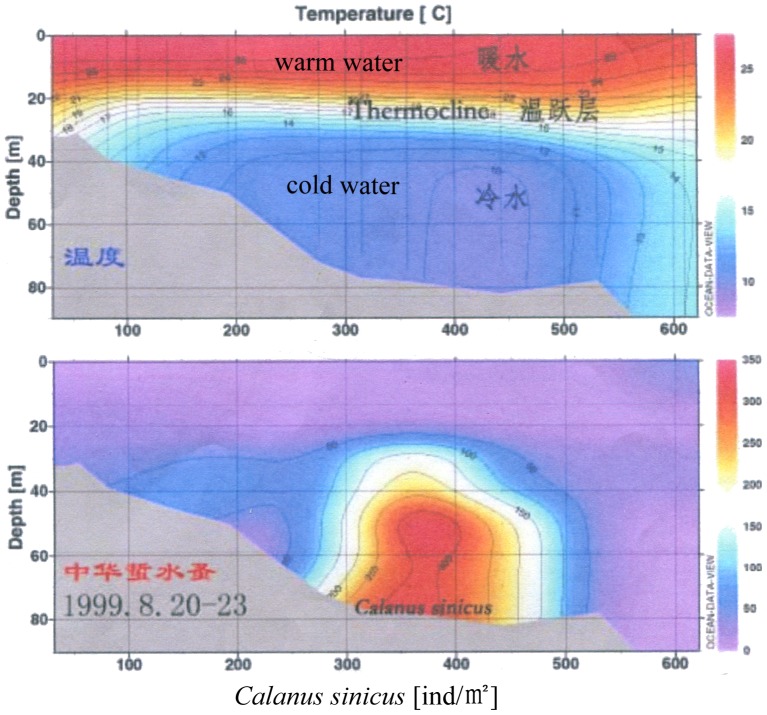
Yellow Sea Cold Water Mass and abundance of *Calanus sinicus* [**11**].

In the central, deeper part of the Yellow Sea, a mass of low-temperature cold water forms in winter by vertical mixing under strong wind. It remains in a bottom layer under the thermocline at 15–30 m depth in summer (Figure 3) [10]. The low-temperature conditions of the Yellow Sea Cold Water Mass protect the cold-water fauna, such as *Calanus sinicus* [11] (Figure 4). A temperate biocommunity dominates the Yellow Sea deeper water and results in a high biodiversity of local bottom fauna in the midlatitude shallow Yellow Sea. It has supported the formation and sustainable development of the north Pacific temperate fauna from the late Pleistocene to the present day. Important fishing activities are carried out in the Yellow Sea. Such is the case of the cold-water fish *Clupea pallasi* (170,000 tons in 1972) and of *Gadus macrocephalus* (50,000 tons in 1958) [7]; these resources have declined seriously in recent years and annual production has decreased to only a few thousand tons [12]. At the same time, recent change in trophic structure of the ecosystem and an increase of fishing effort have resulted in the increased catch of another small cold-water fish—the Pacific sandlance, *Ammodytes personatus*, the annual catch of which in 1999 drastically increased to 500,000 tons. It is a pity that overexploitation decreased the catch to 226,000 tons in 2004, and further to 146,000 tons in 2009 [13].

### The East China Sea

The East China Sea is a marginal sea of the north Pacific, surrounded by China on the continent and by Taiwan, the Korea Peninsula, Japan, and the Ryukyu Islands. It connects with the Sea of Japan through the Tsushima and Korea straits and with the South China Sea through the Taiwan Strait. The sea is 1,300 km long and 740 km wide, covering an area of about 773,700 km^2^. The depth averages about 370 m, with a maximum of 2,719 m. The coastline of the continent is winding and hilly, with many bays, harbors, and islands, of which the Zhoushan Archipelago is well known. More than half of China's islands are in the East China Sea [1], [14].

A number of rivers including the largest river in China, the Changjiang, or Yangtze, pour into the East China Sea. Principal rivers besides the Changjiang are the Qiantang, Oujiang, and Minjiang. Because of this freshwater input, the East China Sea has a vast area of low-salinity water on the shelf in the coastal zone. This area of high biological diversity and productivity includes important fishery resources that have long been exploited [15].

The frontal zone of the Kuroshio Warm Current and the Yangtze brackish water forms a spawning and nursery ground for several species of economically important fishes. The well-known Zhoushan and Shengsi Fishing Grounds of the bighead hairtail, the large and small yellow croaker, and the cuttlefish are all located in this area around the Yangtze River estuary.

### The South China Sea

The South China Sea is one of the largest marginal seas of the western Pacific Ocean, with a surface area of 3.5 million km^2^ and an average depth of 1,212 m. It extends across the tropical and subtropical zones, with an average annual precipitation of 2,000 mm. A large amount of freshwater enters the sea from the Zhujiang (Pearl River) in the north and from the Meikong River in the southwest. The South China Sea connects with the East China Sea, the Pacific Ocean, the Sulu Sea, the Java Sea, and the Indian Ocean through the Taiwan, Bashi, Balabac, Karimata, and Malacca straits, respectively. These straits are all narrow and shallow, except for the Bashi Channel, whose maximum depth exceeds 2,000 m. Consequently, the South China Sea is a semi-enclosed body of water. The bottom topography of the South China Sea is complex. Wide continental shelves appear in the north and in the south; steep slopes in the east and in the west. The 200 m isobath encloses a rhomboid basin elongated to the northeast. The Luzon, Manila, and Nansha troughs are distributed on the east slope, while the Dongsha, Xisha, Zhongsha, and Nansha islands rise from the underwater plateaus. A strong northeastward winter monsoon and a weaker southwestward summer monsoon may lag for up to three months from the south to the north. During the transitional period between winter and summer monsoons, different controlling wind fields may coexist at the sea surface [16].

The complicated geological structure of the South China Sea includes a diversified bottom geomorphology; the continental shelf is distributed along the continent and an island arch. The continental shelf is widest from the northwest to the southwest and narrowest from east to west. Next to the continental shelf in the South China Sea is the stepped continental slope followed by a distribution of deep troughs and canyons. Next to the slope is the central basin with a depth of more than 3,500 m. The South China Sea central basin, located at the slope between the Zhongsha and Nansha islands, is a wide plain with a maximum depth of 5,377 m. In the basin, there are sea dunes and seamounts [1], [4], [16].

The biodiversity of the Chinese cold-water marine biota is not as high as that of the neighboring Japanese waters, and the biodiversity of the warm-water elements is distinctly lower than that of the Philippines-Indonesia-New Guinea triangle—the world center of tropical marine biota. Nevertheless, Chinese marine fisheries production—from both captive fisheries and mariculture—has surpassed that of all other countries since the end of the last century. The serious overexploitation of living resources has decreased the marine biodiversity, collapsed some of the major natural fish and shrimp stocks, and inhibited the sustainable development of marine fisheries. Further efforts should be made to conserve and breed them effectively [8].

Rapid development of coastal agriculture in China has caused an increase in the organic nitrogen and phosphate salt content of coastal waters. Eutrophication of coastal and embayment waters has induced harmful algal blooms of diatoms and dinoflagellates. The red tide hazards in eastern and northern China have resulted in mass mortality of natural and cultured fish or/and shellfish populations and great economic damage [17]–[19].

## Methods

### Research units (laboratories) and survey ships

Before the liberation and the establishment of the People's Republic of China, there was almost no marine science research, and only a few biologists studied fish, invertebrate, and protozoan groups. Research centers were mainly in Qingdao, Shanghai, Xiamen, and Beijing. No research vessel for marine or fishery science existed. Marine biodiversity studies in China began with the establishment of the Marine Biological Laboratory (now Institute of Oceanology) of the Chinese Academy of Sciences (CAS) in 1950.

Specimen collection was carried out by groups of individual scientists along north China coasts from 1951 to 1953 and along the south China coast after 1954. These collections drew mainly from fishing boats, fish markets, and intertidal excursions and thus were limited in number and variety of species. The Bohai Sea Small Yellow Croaker Resource Survey was undertaken in 1952 by the Central Fisheries Research Institute and a joint survey of the Japanese Scomber Yantai-Weihai fishing ground began in 1954.

A new era for marine science and marine biology study began in 1957 with the first cruise of the Chinese Comprehensive Oceanographic Survey Ship, the R/V *Jinxing (Venus)*, of the Institute of Oceanology, Chinese Academy of Sciences (IOCAS). The Comprehensive Oceanographic Survey to the Bohai Sea and northern Yellow Sea (1957–58) was followed by the National Comprehensive Oceanographic Survey in 1958–60 and the China/Vietnam Comprehensive Oceanographic Survey to Beibu Gulf (Tonkin Gulf) in 1959–60 and 1962. Large numbers of specimens of marine fishes and invertebrates were collected and deposited in the Marine Biological Museum of the Chinese Academy of Sciences in Qingdao for taxonomic and biodiversity studies. Other large-scale ocean and marine living resource surveys include the 1981–87 National Coastal Zone and Beach Resources Comprehensive Survey, the 1997–2000 Continental Shelf Environment and Living Resources Survey [8], [20]–[22], and the Xisha and Nansha islands comprehensive oceanographic expeditions in the 1970s and 1985–1990s by the Chinese Academy of Sciences [23].

Generally speaking, the micro- and megaplankton groups of marine biota, as well as macrobenthos and nekton groups, have been better and more intensively sampled than the picoplankton and meio- and microbenthos groups in China seas, particularly on the shelf. In contrast, lack of deep-sea sampling facilities and equipment has prevented sampling and study of deep-sea fauna.

Marine research institutions of China are located mainly in Qingdao, Xiamen, Guangzhou, Shanghai, Hangzhou, and Beijing. Table S1 lists the principal marine science research units in China, while Table S2 provides a list of the scientists working on biodiversity, taxonomy, and systematics of marine biota in China. Table 1 shows the Chinese research vessels that are equipped with biological sampling equipment such as bottom samplers, including grabs, box corers, dredges, and trawl nets.

**Table 1:** Oceanographic and marine science research vessels of China.

Vessel	Affiliation	Gross tonnage	Biological sampling equipment and biological laboratory	Established
Kexue I*	IOCAS	2,579	With biological sampling equipment, experimental laboratory	1980
Kexue III Science	IOCAS	1,220	With biological sampling equipment and marine biological laboratory	2007
Shiyan (Experiment)*	SCSIOCAS	2,560	With biological sampling equipment and marine biological laboratory	2009
Shiyan (Experiment II)	SCSIOCAS	635	With biological sampling equipment and marine biological laboratory	1979
Shiyan (Experiment III)*	SCSIOCAS	2,579	With biological sampling equipment and marine biological laboratory	1980
Xiangyanghong 09*	SOA	2,952	With biological sampling equipment and marine biological laboratory	1978
Xiangyanghong 14*	SOA	2,894	With biological sampling equipment and marine biological laboratory	1980
Xuelong (Snow Dragon)*	SOA	14,997	With biological sampling equipment and marine biological laboratory	1993
Dongfanghong*	COU	3,235	With biological sampling equipment and marine biological laboratory	1995
**Yanping II	FIO	386	With biological sampling equipment and marine biological laboratory	1995
**Beidou (Polaris)	YSFRI, CAFS	980	Fishery Research Vessel [G O Sars type]	1983

**Notes:**

* Oceangoing research vessel (multidisciplinary oceanographic).

IOCAS, Institute of Oceanology, Chinese Academy of Sciences, Qingdao.

SCSIOCAS, South China Sea Institute of Oceanology, Chinese Academy of Sciences, Guangzhou.

SOA, State Oceanographic Administration.

COU, China Ocean University, Qingdao.

FIO, Fujian Institute of Oceanology, Xiamen.

** YSFRI, CAFS, Yellow Sea Fisheries Research Institute, Chinese Academy of Fisheries Science (with trawl net and fish biology laboratory).

### Taxonomy and biodiversity studies

A great number of marine biological specimens (fishes, invertebrates, protozoans, micro- and macroalgae) have been collected by the Marine Biological Laboratory and the IOCAS marine biodiversity and resource research group along the China coasts since 1950. Taxonomic experts have identified specimens of various groups of phyto- and zooplankton and zoobenthos, as well as pelagic and demersal fishes, to species category by taxonomy and biodiversity studies and for analysis of faunal and floral communities and ecosystems. Research fields cover the major groups of marine biota, including micro- and macroalgae (Cyanophyta, Diatomeae, Phaeophyta, Rhodophyta, Chlorophyta), seagrasses and mangroves; the protozoan kingdom including phyla Ciliophora, Foraminifera, Radiolaria, Dinozoa; invertebrates from the phyla Porifera, Cnidaria, Trematoda, Annelida (Polychaeta), Bryozoa, Entoprocta, Phoronida, Brachiopoda, Sipuncula, Echiura, Mollusca, Crustacea, Chaetognatha, Hemichordata, Urochordata (Tunicata), Chordata; as well as the subphyla Cephalochordata and Vertebrata, including fish classes and Mammalia.

## Results

### Biodiversity of marine life of the China seas

**Figure 5:**
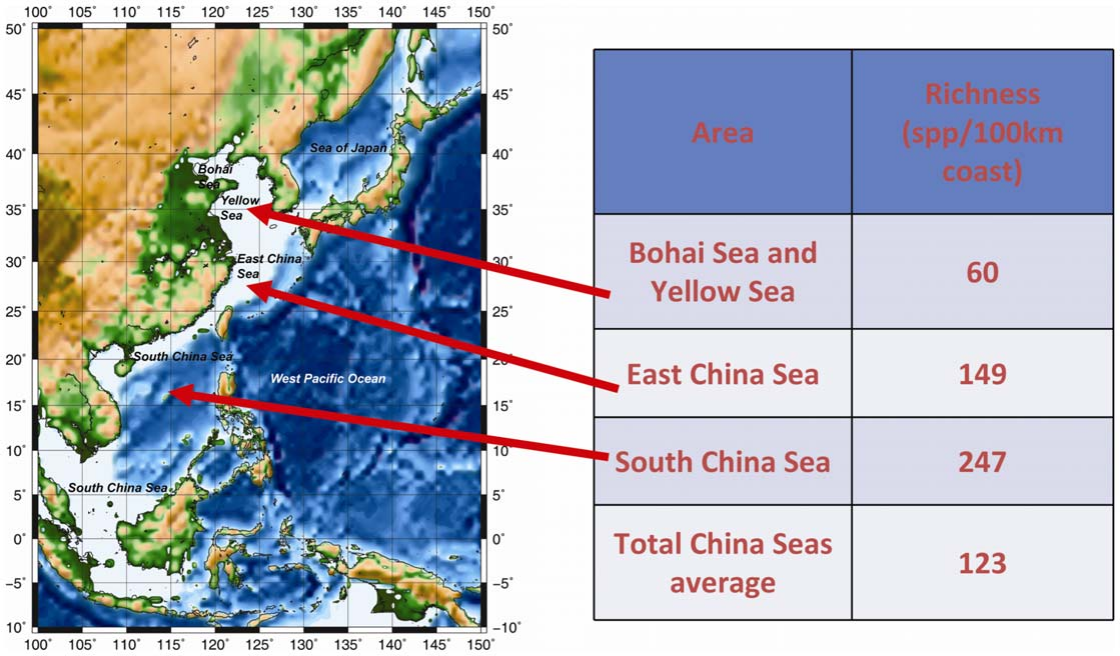
The number of species in China's seas increases distinctly from the north to the south—from high to low latitude.

The seas surrounding mainland China and its southern islands and reefs span 38 degrees of latitude (3°–41°N) from the tropical to the warm-temperate climate zones and include the widest continental shelf in the Eastern Hemisphere. Under the influence of the strong Kuroshio Warm Current, the South China Sea Warm Current, and the Taiwan Warm Current, the water temperature of the East and South China seas is comparatively high, warmer than 14–16°C in coastal areas in winter. The marine flora and fauna of the China seas are rich in warm-water species comprising tropical and subtropical elements of the Indo-West Pacific Biotic Region with high dominance of some endemic and economically important species, mainly endemic to the East China Sea and neighboring waters. The tropical Indo-Malaysian biotic elements that are transported by these warm currents originated in the south, while the cold-water species came from the north and dominate deeper parts of the Yellow Sea Cold Water Mass under the thermocline at the 15–30 m layer in summer. Thus, the China seas are characterized by high biodiversity (Table 2), including particularly rich tropical and subtropical elements in the East and South China seas and temperate biota in the Yellow and Bohai seas. However, fewer cold-water species are found in the Yellow Sea than in waters off northern Japan, which are dominated by the strong Oyashio Cold Current, while the number of tropical elements found in the South China Sea is less than that recorded in the Luzon-New Guinea-Indonesia triangle area—the center of tropical Indo-West Pacific Biota. Low temperatures in winter have limited the survival of warm-water species, resulting in low biodiversity. Therefore, among a total of 22,629 Chinese marine species, only 1,607 species live in Yellow Sea (Table 3). It is noteworthy that the strong Tsushima Current brings some warm-water species and flows along the west coast of Honshu in the Sea of Japan north to the Tsugaru Strait [8]. Consequently, the number of warm-water species in the eastern Sea of Japan is much more than in the western Russian Siberian waters, where the marine fauna is cold temperate, while the majority of the eastern Japanese fauna is warm temperate. In addition, the number of species in China's seas increases distinctly from the north to the south—from high to low latitude (Tables 4 and 5, Figure 5).

**Table 2:** Estimated numbers of described and undescribed species per taxon, along with information on the number of endemic and introduced species, and number of experts and identification guides.

Taxonomic group	Number of species	Possible increase of species	Number of endemic species	Number of introduced species	Number of experts	Number of identification guides
**Domain Prokaryota Kingdom Bacteria**	**264**	300+62	12	Uncertain	5	1
**Domain Eukaryota**	**22,365**				132	196
**Kingdom Chromista**	**1,807**		323	1	15	29
Phaeophyta	260	38			2	2
Diatomeae	1485	125			8	16
**Kingdom Fungi**	**151**		Uncertain	Uncertain		0
**Kingdom Plantae**	**792**		142	1	6	11
Chlorophyta	163	48	17		2	1
Rhodophyta	569	38	123		2	7
Angiospermae	58				2	2
**Kingdom Protozoa**	**2,897**				10	8
Dinomastigota (Dinoflagellata)	302				2	
Foraminifera	1495	∼380	0	0	1	4
Radiozoa	594	∼48	0	0	3	1+1
Ciliophora	503	∼2500			4	3
**Kingdom Animalia**	**16,718**				90	132
Porifera	190	∼20	3	1	1	9
Cnidaria	1,422	40∼50	202		11	7
Platyhelminthes (Trematoda)	535	0	308	Uncertain	2	3
Mollusca	3,914	∼650	206	3	6	29
Annelida (Polychaeta)	1,065	∼15	104		3	3
Crustacea	4,320	∼1000	411	7	18	27
Bryozoa	568	∼900	Uncertain	Uncertain	2	2
Echinodermata	588	56	40	0	1	4
Urochordata (Tunicata)	139	3	7	2	4	7
Other invertebrates	449	Nematoda ∼800	Uncertain	Uncertain	16	11
Chordata	3,532		71	Uncertain		
Cephalochordata	4					
Vertebrata Pisces	3,213	77			12	19
Aves	249	∼10			5	6?
Mammalia	41	3			3	2
Other vertebrates	25				14	11
**SUBTOTAL**	**22,629**	**∼7,113**	∼1,969	∼15	137	197

**Table 3:** Number of different kinds of cold-water and warm-water species of invertebrates and fishes recorded in the Yellow Sea [8].

Fauna	Invertebrate	Fish	Total
Cold-water species			
North-hemisphere temperate species	33 (2.5%)	2	35
Amphi-boreal species	37 (2.8%)	2	39
North Pacific temperate species	41	9	50
Amphi-Pacific temperate species	59	5	64
Northwest Pacific temperate species (Russia–Japan–N. China (Yellow Sea))	100	29	129
Yellow Sea–Japan warm-temperate species	158	74	232
Species endemic to Yellow Sea	102 (7.9%)	8	110
Warm-water species			
China seas warm-water species (Bohai Sea, Yellow Sea, East China Sea, and South China Sea)	92 (7.1%)	10	102
Sino-Japan warm-water species	194	55	249
West Pacific warm-water species	187	37	224
Indo-West Pacific warm-water species	164	85	249
Cosmopolitan species	52	0	52
Others	67	5	72
Total	1,286	321	1,607
Total cold-water species	531	129	660
Percentage of cold-water species (%)	41.3	40.2	41.1

**Table 4:** Number of macrobenthic invertebrate species in China seas [8].

Taxonomic group	China seas	Bohai and Yellow seas	East China Sea	South China Sea
PORIFERA	199 spp.	10 spp.	30 spp.	140 spp.
CNIDARIA	1005	50	281	707
-Hydrozoa	245	45	132	130
-Antipatharia	41	0	1	41
-Octocorallia	328	4	95	227
-Scleractinia	394	1	53	309
ANNELIDA POLYCHAETA	1065	367	376	643
MOLLUSCA	3854	481	1034	2391
-Chaetodermomorpha	1	1	0	0
-Neomenionorpha	1	0	0	1
-Polyplacophora	47	14	9	19
-Scaphopoda	56	2	20	36
-Gastropoda	2492	765	646	1430
-Bivalvia	1132	185	319	802
-Cephalopoda	125	14	40	103
ARTHROPODA (CRUSTACEA)	3008	409	703	2150
-Thoracica	198	22	82	163
-Leptostraca	1	1	0	0
-Stomatopoda	104	5	30	87
-Mysida	103	29	44	86
-Gammaridea	373	92	102	292
-Caprellidea	18	13	6	10
-Isopoda	174	31	38	133
-Dendrobranchiata	134	11	42	105
-Caridea	409	49	85	324
-Stenopodidea	3	0	1	3
-Astacidea	17	0	4	7
-Axiidea & Gebiidea	54	13	9	13
-Palinuridea	45	0	24	29
-Anomura	299	24	78	130
-Brachyura	1073	122	340	771
-Xiphosura	3	0	0	3
-Pycnogonida	10	5	2	5
BRYOZOA	568	116	231	402
ECHINODERMATA	588	54	175	427

**Table 5:** Species richness in China seas by kilometer of coast.

Region or habitat	Species richness (species/100 km coastline)
Average of all China seas	123
Bohai Sea and Yellow Sea	60
East China Sea	149
South China Sea	247
Hainan Island	268
Xisha Islands	2,700 species/10 km coastline*
Nansha Islands	4,642/100 km (6,500 species/140 km coastline)

* IOCAS studied less than 10 km of coastline in the Xisha Islands.

### Characteristics of marine fauna and flora of the China seas

#### 1.Tropical fauna of the South China Sea

Scleractinian corals and coral reef-communities in the southernmost coast of Hainan and South and East Taiwan Island are well developed to form fringing reefs with rich benthic communities; while the coral reef atolls in the southern and mid part of the South China Sea Islands—the Dongsha (Pratas), Xisha (Paracels), and Nansha (Spratlys) islands—are basically tropical (stenothermal).

In waters adjacent to southern to southernmost Hainan Island and south and east Taiwan coasts, tropical species of scleractinian coral and reef communities increased, and well-developed fringing coral reefs were recorded. Tropical elements including six species of tridacnid mollusks and two species of Fimbridae; in addition, *Pedum spondyloidesum, Anomiostreum corliophyla, Codakia tigerina, Ovula ovuma*, and species of Cypraeidae and Conidae have been recorded. Echinoderms from tropical South China Sea include, for example, *Toxopnustas pilealus, Actinopyga mauritiana, Holothuria arta, Culcita novaeguineae, Linckia laevigata*, and *Stichopus chloronotus*. The following species of coral-dwelling decapod crustaceans are commonly found in waters off southernmost Hainan Island: *Alpheus lottini* and alians, *Synalpheus demani*, and congener *Coralliocaris* species, *Jocaste* species, *Saron mammoratus, Thor amboinnensis, Th. paschalis*, the coral reef crab *Trapezia* species, *Tetralia* species, and *Thalamita* species. In addition, some typical Indo-Malaysian tropical species of fishes and other invertebrates are also commonly found in Hainan.

Long-term investigations have proved that some typical tropical stenothermic species have not been found in Hainan waters. Species not found include the echinoderms because southernmost Hainan and south and east Taiwan coasts are located at the northern limit of coral reef distribution. The reef structure and reef communities are not typical, and certain typical species such as *Tubipora musica, Heliopora aerulia, Acropora echinata, A. palifera, Stylophora* species, and *Seriatopora* species have not been found along the Hainan coast. Other typical species not recorded here are *Heterocentrotus mommillatus, Metalia dicrana, Actinopyga ananas, Thelenota ananas, Choriaster granulatus, Linckia multifora*, *Ophiothrix trilineata*, etc.; the mollusks *Conus leopardus, C. legatus, C. tenuistriatus, Cassis cornuta, Strombus erythrinus, S. dentatus, Malleus regula, Fimbria fimbria, F. soverbii, Tridacna gigas, Hippopus hippopus*; the crustaceans *Thalamita coeralepes, T. tenuis, T. demani*, and *Percnon* species, etc. These species are main components of the Indo-Malaysian Tropical Faunal Region and indicate that Hainan is really at the edge of the tropical biotic region. The shelf of the northern South China Sea includes abundant distributions of decapod crustaceans and bivalve mollusks. Among the 146 species of penaeid shrimps and 126 alpheid shrimps recorded in the South China Sea, the common and dominant species are *Atypopenaeus compressipes*, *Metapenaeus affinis*, *M. ensis*, *M. jouneri*, *M. moyebi*, *M. intermedia*, *Parapenaeopsis hardwickii*, *P. tenella*, *P. cornuta*, *P. chinensis*, *P. hungerfordii*, *Trachysalambria curvirostris*, *T. longipes*, *T. albicomes*, and the solenocerids *Solenocera crassicornis*, *S. koelballi*, *S. albicristatus*, *Metapenaeopsis barbata*, and *M. sinica*. Additionally, more than 15 species of *Merepenaeopsis* are commonly distributed in the shelf sea, while in the outer shelf and the marginal waters, eight species of *Parapenaeus* and two species of *Penaeopsis* are recorded. High abandance of these shallow-water penaeid shrimps supported a large and strong shrimp fisheries in the shelf sea of southern and eastern mainland China [24], [25].

#### 2. Subtropical fauna of the East China Sea

**Figure 6:**
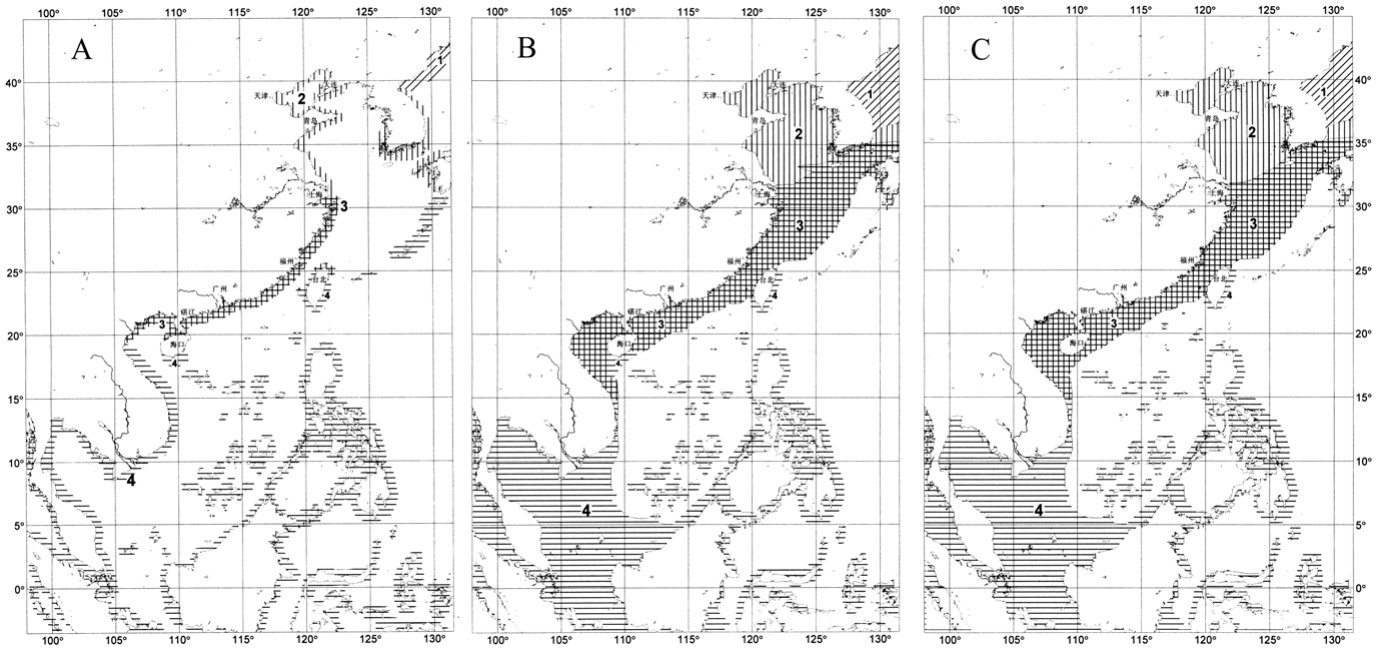
Marine fauna and flora of the China seas. Shaded area 1 is the North Pacific Temperate Biotic Region, Far Eastern Cold-temperate Subregion. Shaded area 2 is the North Pacific Temperate Biotic Region, East Asia Warm-temperate Subregion. Shaded area 3 is the Indo-West Pacific Warm-water Biotic Region, China-Japan Subtropical Subregion. Shaded area 4 is the Indo-West Pacific Warm-water Biotic Region, Indo-Malaysian Tropical Subregion. 6A. Distribution of Phyto-benthic Flora; 6B. Distribution of Zoo-benthic Fauna; 6C. Distribution of Marine Fish Fauna.

Marine fauna and flora of the East China Sea consist mainly of warm-water elements belonging to the Indo-West Pacific Biotic Region (Figure 6). They are characterized by high biodiversity. The tropical and subtropical biotic elements transported by the Kuroshio and other warm currents from the south include many species common to the South China Sea, and some are endemic to the China seas (Bohai–South China Sea) having large populations with high economic value in local fisheries. They are dominantly distributed in the eastern and southern shelf seas of mainland China (East and South China seas) (also in Japan) in high abundance. These species include, among others, the well-known large yellow croaker *Larimichthys croacea, the* small yellow croaker *Larimichthys polyactis*, the Chinese shrimp *Fenneropenaeus chinensis*, the Chinese Maoxia (Akyami) *Acetes chinensis*, the shrimps *Palaemon gravieri, P. tenuidactylus*, *Exopalaemon annandalei* and Chinese krill *Pseudeuphausia sinica*; and the bivalve *Trigonothracia jinxingae*. Large populations of these endemic species together with the other tropical Indo-Malaysian fauna distributed abundantly in shelf areas have supported a strong fish and shrimp fishery of the East China Sea.

It was found that many warm-water species of invertebrates are dominantly distributed in the shelf of the South and East China seas, such as the penaeid shrimps *Metapenaeopsis dalei, M. barbata, Parapenaeus fissuroides, Solenocera koelbelli, M. tenella, M. provocatoria longirostris, Solenocera alticariata, Parapenaeopsis hardwickii, P. tenella, Trachysalambria curvirostris*, and *Metapenaeus joyneri* and the caridean shrimps *Palaemon grvieri, Plesionika izumiae*, *Procletes laevicarina*, etc. The rich resources of these species have supported a strong penaeoid shrimp fishery since the late 1980s and made great contributions to local fisheries together with the above-mentioned endemic species of China shelf seas [26], [27].

In coastal shallow waters, low-salinity species such as Exopalaemon carinicauda, E. annandalei, Palaemon tenuidactylus, Leptochela graciis, Alpheus distinguendus, and A. japonicus are dominant.

#### 3. Temperate fauna of the Yellow Sea

Marine fauna of the Yellow Sea (where the summer bottom-water temperature does not exceed 10°C in the north and 12°C in the south) belong to the North Pacific Temperate Biotic Region [24], [25]. They are dominated by cold-water species represented by the ophiurids *Ophiura sarsii vadicola* and *Ophiopholis mirabilis*; the bivalves *Thyasira tokunagai, Clinocardium californiense, Nucula tenuis*, and *Musculus nigra*; the decapod crustaceans *Oregonia gracilis*, *Pagurus ochotensis, P. pectinatus, Heptacarpus camtschaticus, Eualus gracilirostris, E. spathulirostris and Crangon hakodatei, C. uritai*; and the sponge *Suberites domuncula*. Most of these species are common to the Yellow Sea and northern Japan (and sometimes also to Russian Siberian seas) [8].

For most dominant cold-water benthic invertebrate species mentioned above, the distribution range is limited to the Yangtze River mouth, between the Yellow Sea and the East China Sea [26], although the range of some species extends southward to the Zhoushan Archipelago in the northern part of the East China Sea. The boundary between the tropical-subtropical biotic region and the warm-temperate biotic region extends roughly from the Yangtze River Estuary eastward to Niigata, northern Honshu, Japan, and north to Cheju Island and the Korea Strait. The boundary between the China–Japan Subtropical Subregion and the Tropical Southern South China Sea Subregion extends roughly from mid- or southern Vietnam to Amami Ohshima Island, north of Okinawa, Japan, through the southernmost sea area off Hainan Island and the southeast coast of Taiwan. There, scleractinian corals and coral communities are dominant and rich in species.

The marine biota of the shallow water of Yellow Sea are characterized by a small number of eurythermic warm-water species together with some cold-water (temperate) species that can adapt to lower temperatures in winter or higher temperatures in summer. Although the number of species is comparatively small, they represent large populations that have large economic value in local fisheries.

In his work on the biogeographic characteristics of the marine biota of the northern Pacific waters, Briggs [27] recognized a cold-temperate fauna inhabiting the western North Pacific. He named the region the “Oriental Province,” of which the southern portion includes the Yellow Sea, the central portion is the Sea of Japan, and the northern portion lies along the Pacific coast of northern Honshu. He wrote that the tip of the Korean Peninsula supports a warm-temperate fauna and cited Ushakov [25] that “an appreciable number of the polychaete species characteristic of the Far Eastern seas of the USSR were also found in the northern part of the Yellow Sea.” He noted that “the resident marine fish fauna of the Yellow Sea apparently consists mainly of cold-temperate species that are also found in the northern part of Sea of Japan.” The present biodiversity study group argues that the Yellow Sea fauna is warm temperate, being part of the North Pacific Temperate Faunal Region, Eastern Asia Subregion [28] and not affiliated with the Indo-West Pacific warm-water region. Based on this fact, we conclude that the cold-water communities are distributed in high and predominant abundance evidencing high species diversity in deeper areas. The presence of the shallow and coastal water amphiboreal and circumpolar species, such as the well-known abnormal echinoid *Echinocardium cordatum*, the phyllopod crustacean *Nebalia bipes*, and the bivalve mollusk *Mya arenaria*, in the Yellow Sea [8] indicates that both cold- and warm-water species coexist in the shallow waters. A few eurythermal warm-water species, mainly shrimps, crabs, and bivalves, are abundant in shallow waters of the Yellow Sea and Bohai Sea, and almost all are fishery resources of economic importance. In addition, more warm-water (subtropical) species and some cold-water (temperate) species coexist along the south and southeastern coasts of Korea Peninsula [8], [32].

#### 4. Plankton communities

**Figure 7:**
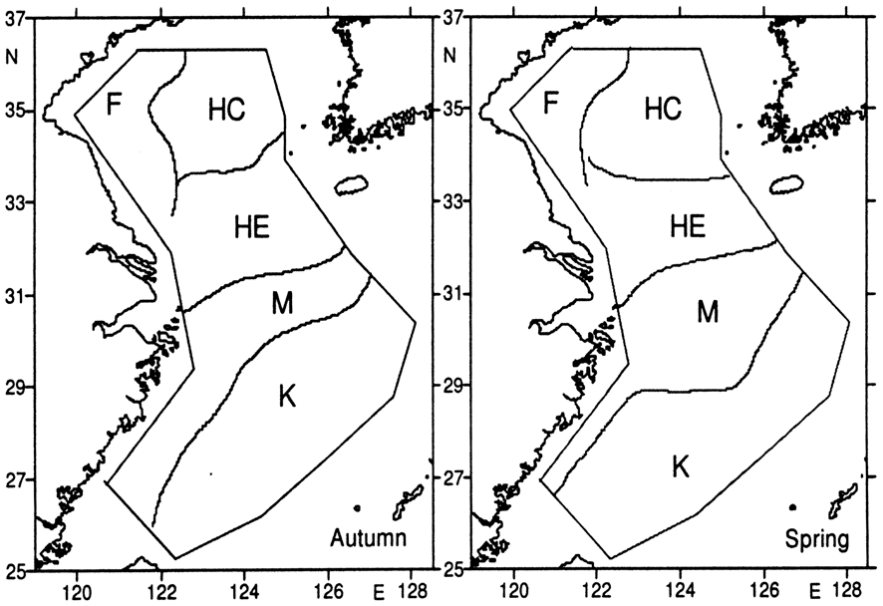
Distributions of zooplankton communities in the East China Sea and Yellow Sea. K - East China Sea Shelf High-Temperature and High-Salinity Community, M – East China Sea Coastal Mixed Community, HE – Yellow Sea-East China Sea Mixed Community, F- Yellow Sea Coastal Community, HC - Central Yellow Sea Community.

Plankton plays an important role in trophic energy flow and material transfer in the marine ecosystem and food web (food chain) and in biogeochemical circulation. Plankton communities in the China seas have been preliminarily described and discussed since the 1960s. Cold- and warm-water plankton communities dominated the Yellow and northern East China seas and play important roles in maintaining the trophic relationship and biological production of the ecosystem. In addition, their role in the sustainable use of resources has been recognized, analyzed, delineated, and described. Further study of plankton communities of the Yellow and East China seas indicates that the structure and distribution of the plankton community reflects the oceanographic characteristics of the inhabiting seas. The following five communities have been distinguished by Zuo Tao [29] based on new data obtained recently (Figure 7) (Table 6).

**Table 6:** Composition of indicator species in plankton communities of Yellow Sea and East China Sea in spring and autumn.

Community	Autumn	Spring
**East China Sea (K)**	*Rhincalanus cornutus (100, 95)*	*Sagitta enflata(100,97)*
**Outer Shelf**	*Eucalanus subtenuis(100, 94)*	*Eucalanus subtenuis(76,99)*
	*Lucicutia flavicornis(89, 100)*	*Doliolum denticulatum(100,75)*
	*Sagitta neglecta(100, 86)*	*Lucifer intermedius(95,79)*
	*Undinula darwinii (94, 90)*	*Eucalanus pseudattenuatus(66,100)*
	*Eucalanus subcrassus(100, 85)*	*Aglaura hemistoma(67,97)*
	*Nannocalanus minor(94, 89)*	*Rhinocalanus cornutus(71,87)*
	*Scolecithrix danae(83, 99)*	*Acrocalanus gibber(61,100)*
	*Eucalanus attenuatus(83, 97)*	*Corycaeus speciosus(62,100)*
	*Clausocalanus arcuicornis (89, 87)*	*Temora stylifera(62,100)*
	*Rhincalanus nasutus(78,96)*	*Pterosagitta draco(57,100)*
	*Pleuromamma robusta(83,89)*	*Abylopsis tetragona(83,67)*
	*Temora discaudata(78,95)*	*Diphyes chamissonis(57,94)*
	*Sagitta robusta(78, 85)*	*Pleuromamma gracilis(52,99)*
		*Physophora hydrostatica(57,86)*
		
**East China Sea (M)**	*Sagitta bedoti(85,81)*	*Euphausia pacifica(81,70)*
**Coastal**	*Euconchoecia aculeate(69,79)*	*Phialidium chengshanensis(56,97)*
	*Pseudeuphausia sinica(54,63)*	*Pleurobrachia globosa(56,74)*
		*Euchaeta plana(100,54)*
		*Muggiaea atlantica(100,64)*
		*Solmundella bitentaculata(69,55)*
		*Acanthomysis longirostris(56,52)*
		*Pseudeuphausia sinica(50,40)*
		*Dolioletta gegenbauri(62,46)*
		
**Yellow Sea-East China (HE)**	*Acanthomysis sinensis(54,44)*	*Pseudeuphausia sinica(88,52)*
	*Sarsia jiponica (100,5)*	*Labidocera euchaeta(75,54)*
	*Acartia pacifica(89,12)*	*Acanthomysis longirostris(44,30)*
	*Euconchoecia aculeate(88,2)*	*Paracalanus parvus(100,61)*
	*Euchaeta concinna(100,1)*	*Acartia bifilosa(69,85)*
		
**Central Yellow Sea (HC)**	*Themisto gracilipes (92,68)*	*Oithona similes (100,93)*
	*Calanus sinicus (92,61)*	*Sagitta crassa (100,49)*
	*Euphausia pacifica (58,58)*	*Themisto gracilipes(100,45)*
	*Sagitta nagae (92,49)*	
		
**Yellow Sea (F)**	*Labidocera euchaeta(91,93)*	*Centropages mcmurrichi (100,88)*
**Coastal**	*Acartia pacifica(91,82)*	*Proboscidactyla flavicirrata (71,76)*
	*Clytia hemisphaerica(55,100)*	*Ophiopluteus larva (57,87)*
	*Beroe cucumis(64,79)*	
	*Pleurobrachia globosa (64,76)*	
	*Muggiaea atlantica (55, 78)*

1. Yellow Sea Coastal Community (F) – indicator species: *Labidocera euchaeta* (Autumn), *Centropages mcmurrichi* (Spring)
2. Central Yellow Sea Low-Temperature Community (HC) – indicator species: *Themisto gracilipes, Calanus sinicus, Euphausia pacifica, Sagitta* species (Autumn), *Calanus sinicus* and *Euphausia pacifica*
3. East China Sea Outer Shelf High-Temperature High-Salinity Community (K) – indicator species: *Rhinocalanus cornutus, Pterosagitta draco*, many stenothermic species
4. Yellow Sea–East China Sea Mixed Community (HE) with many temperate coastal low-salinity species – indicator species: *Acanthomysis longirostris, Sarsia Japonica, Acartia pacifica*
5. East China Sea Coastal Mixed Community (M) with many widely distributed warm-water species – indicator species: *Sagitta bedoti, Pseudeuphausia sinica*

### Changes in marine biodiversity in various sea areas and habitats

In the following paragraphs, results of long-term monitoring studies in (1) a tidal flat in a semi-enclosed bay, (2) a cold-water ecosystem, (3) the tropical coral reefs, (4) mangrove forests, (5) seagrass fields, and (6) shrimp stock enhancement practice are synthesized to show the impact of global climate change and human activities.

#### (1) Change of benthic fauna biodiversity in a semi-enclosed bay—a muddy tidal flat in the Yellow Sea

Semi-enclosed embayments are well known for their rich biodiversity and high productivity. Marine environments, ecology, and living resources of the main embayments around China have been studied. These include the Jiaozhou Bay and the Sanggou Bay in Yellow Sea [30]–[33], the Xiangshan Harbor in East China Sea [34], the Quanzhou Bay [35] and Xiamen Bay [36], [37] in Taiwan Strait, the Daya Bay [38] and Sanya Bay [39] in northern South China Sea [40]. Long-term monitoring indicates that data obtained by intensive sampling are valuable for biodiversity analysis.

Great changes in species composition, biodiversity, and abundance have been found in coastal industrial areas impacted by coastal exploitation, eutrophication, pollution, and habitat loss. Species composition and abundance of intertidal benthic fauna and flora of a sandy mud tidal flat in Cangkou on the east coast of Jiaozhou Bay on the Yellow Sea was monitored along a depth gradient between 1935 and 1988 (Table 7). A total of 63 species of benthic invertebrates dominated by Mollusca and Crustacea were found in 1957 [41]; while 141 species (52 Crustacea, 41 Polychaeta, 40 Mollusca, and 3 Echinoderms) were recorded in 1963–64 [32]; and 164 species were found in 1967–68 [30]. Unfortunately, only seven species were found in the 1980s and no living benthic animals were found at the same locality after 1989. The biodiversity was also greatly changed after the 1970s because of heavy industrial pollution and the great change in the intertidal environment due to coastal exploitation [30]–[32].

**Table 7:** Taxonomic composition and species number found in intertidal communities of the Cangkou mud flat, 1935–1988.

Species groups	1935–1936	1947	1950	1957	1963–1964	1974–1981	1987–1988
Coelenterata	1		3		2	1	0
Polychaeta	4	1	9	12	41	2	0
Mollusca	14	11	20	18	40	11	0
Crustacea	12	6	11	28	52	13	0
Brachiopoda	1	2	1	1	1		0
Echinodermadata	1	2	4	2	3	1	0
Protochordata	1		6	2	2	1	0
**Total**	**34**	**22**	**54**	**63**	**141**	**29**	**0**

#### (2) Global climate change and biodiversity of cold-water fauna of the Yellow Sea

As mentioned earlier, most part of the Yellow Sea is less than 100 m deep. The marine fauna of this area consists of three major elements:

1. the cold-water species of North Pacific temperate fauna, most of which inhabit central deeper parts of the Yellow Sea more than 40–50 m deep (species such as those mentioned above);
2. the eurythermal warm-water species that dominate the shallow and coastal waters, some of which are endemic to the China seas and characterized by large populations (e.g., the shrimps *Fenneropenaeus chinensis* and *Acetes chinensis*, and the Euphausid *Pseudeuphausia sinica*); and
3. the warm-water elements that dominate offshore deep waters of the East China Sea and the southernmost part of the Yellow Sea affected by the Taiwan Warm Current and the Yellow Sea Warm Current [24].

Available data and information indicate that the cold-water faunal elements of the Yellow Sea are now declining or even becoming locally extinct under the impact of global climate change (warming). Such is the case of the tellinid bivalve *Peronidia zyonoensis*, which has been recorded in the East China Sea since about 6 B.C. [42], but it is now locally extinct in this area, while living individuals can still be found in colder waters in the northern Sea of Japan (Russian Far East) and the east (Pacific) coast of Honshu, Japan.

**Figure 8:**
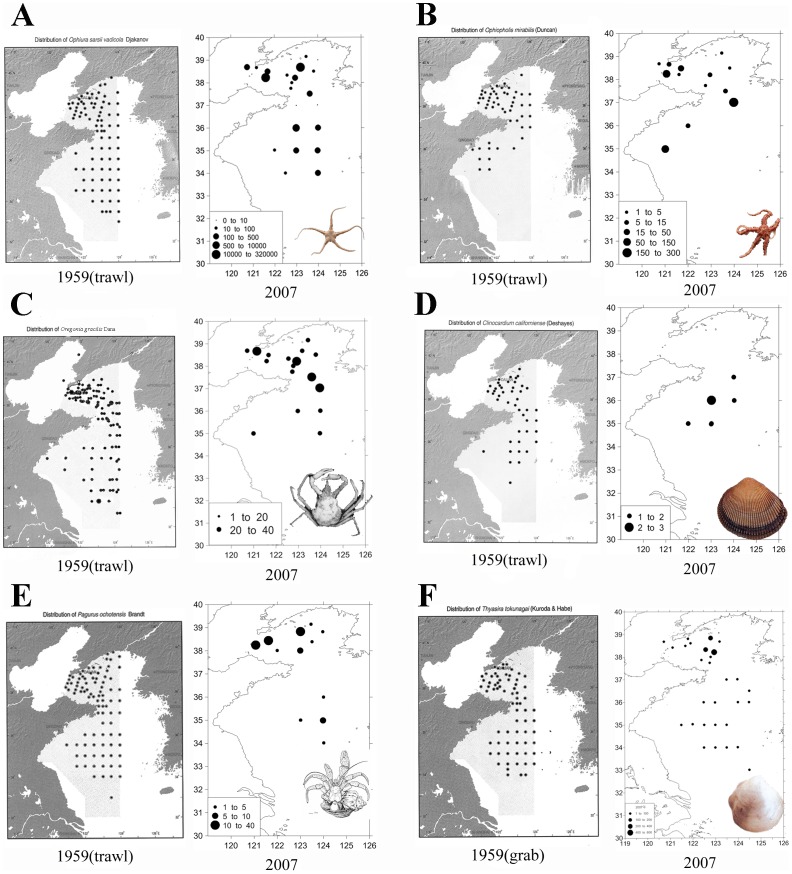
Distribution and abundance of certain cold-water benthic species in the Yellow Sea (sampled in 1959 and 2007). A. *Ophiura sarsii vadicola* Djakonov, 1954; B. *Ophiopholis mirabilis* (Duncan, 1879); C. *Oregonia gracilis* Dana, 1851; D. *Clinocardium californiense* (Deshayes, 1857); E. *Pagurus ochotensis* Brandt, 1851; F. *Thyasira tokunagai* Kuroda and Habe, 1951.

Great changes in structure of some benthic biocommunities have been found that may be the result of global changes in climate and water temperature. Recent investigations have revealed that populations of some cold-water species previously dominating the area, such as *Pagurus ochotensis, Oregonea gracilis*, and *Pandalus prensor*, are now in decline and are becoming rare in the Yellow Sea. This decline indicates that significant changes in species composition and abundance of certain cold-water species have occurred (Figure 8).

#### (3) Monitoring and conservation of coral reef ecosytems

Coral reef community surveys were carried out by IOCAS starting in the late 1950s and continuing from the 1970s through the 1990s. The Sino-Soviet marine biological investigation survey to Hainan Island (1958–1960) and the Sino-Germany Hainan Marine biological Investigation (1990, 1992) have collected and studied many tropical invertebrate and fish and macro-algal species. These surveys recorded as many as 130 species of scleractinian (reef-building) corals, 694 species of decapod crustaceans (including 86+30 = 146 alpheid shrimps and 61 pontoniid shrimps), 485 species of bivalve mollusks, 514 species of gastropod mollusks, 292 species of echinoderms and 298 species of polychates. The fish species amounted to 909. To date, 4,892 species of invertebrates and fish, including 6 species of Tridacnidae and many tropical coral-reef-inhabiting species, have been recorded by my group. The boundaries of the tropical fauna and flora were found from mid- (or southern) Vietnam across southernmost Hainan Island, southern and eastern Taiwan Island northeastward to the north Ryukyu Islands Archipelago. The area includes Xisha, Dongsha, Zhongsha, and Nansha islands in China and Amami Ohshima and Okinawa in Japan. The coral reef ecosystems in the China seas exhibit high biodiversity. More than 200 species of scleractinian corals live in the northern South China Sea, about two-thirds of the world species. They are distributed mainly along the coasts of southern parts of Hainan and Taiwan and the Dongsha, Xisha, and Nansha islands. Table 8 shows the number of species in different parts of the China seas [43].

**Table 8:** Number of genera and species of scleractinian corals recorded from several localities of the China seas [47].

	Nansha Islands	Xisha Islands	Dongsha Islands	Huangyan Islands	Hainan	Taiwan	Hong Kong	Guangdong and Guangxi	Fujian
**Genera**	50	38	27	19	34	58	21	21	
**Species**	200	127	70	46	110	230	50	45	>10

The coral reef structure is mainly atolls in Nansha, Xisha, and Dongsha islands and fringing reefs along the coasts of Hainan and Taiwan islands. Coral reefs along the mainland provinces consist of scattered polyps of individual species not forming a fringing reef. High biodiversity was recorded from the Nansha Islands in the southern part of the South China Sea. A total of 6,500 marine species, including 200 species of reef-building corals, have been recorded from the atolls of the Nansha Islands [23].

High biodiversity has been observed and described in the southernmost area of Hainan Island, Luhuitou, Sanya City [44], but changes in the composition and distribution of corals along a vertical gradient within the reef were observed between 1958 and 1990–1992 [45]. In contrast, no great changes in species composition within this time frame were observed in the subtidal zone communities [44], [45] (Table 9).

**Table 9:** Changes in species composition along the different sections of the Coral Reef in Luhuitou, Sanya, Hainan Island, between 1966 and 1991.

			1966 [50]		1991 [51]
	Coral debris		No living corals		120 m
**Reef flat**	*Goniastrea* Zone	Boulder Negroheads	*Goniastrea aspera*		*Goniastrea yamanarii* 150 m
			*Goniastrea yamanarii*		*Porites lutea* 180∼200 m
			*Favia speciosa*		*Fungia fungites*
					250 m
	*Montipora* Zone	Moat	*Montipora ramose*		
			*Goniastrea aspera*		*Fungia fungites* 250∼300
			*Goniastrea yamanarii*		*Montipora ramosa*
			*Favia speciosa*		*Pocillopora lingulata*
			*Acropora pulchra*		*Platygera rustrica*
			*Fungia fungites*		
			*Fungia sp.*		
			*Porites lutea*		
					330 m
	*Acropora* Zone	Shingle Rampart		No living corals	
		Upper Zone	*Acropora* Zone, Upper	*- - - - - - - - - - - - - - - - - - - - - - - - - - - - - - - - - - - - -* 380 m	
			*Acropora corymbosa*	*Psammocora contigua*	*Acropora corymbosa*
			*A. surculosa*	*Pavona frondifera*	*A. surculosa*
			*A. prostrate*	*P. (Polyastra) minikoiensis*	
			*Pocillopora brevicornis*	*Porites lutea*	
			*P. danae*	*P. matthaii*	
			*P. damicornis*	*P. pukoensis*	
			*Acropora armata*	*Favia speciosa*	
			*A. brueggemanni uncinata*	*F. palauensis*	
			*A. conferat*	*Goniastrea pectinata*	
			*A. nasuta*	*Goniastrea yamanarii*	
			*A. pulchra*	*Platygyra crosslandi*	
			*A. pulchra stricta*	*P. rustica*	
			*Montipora fruticosa*	*Hydnophora contignatio*	
			*M. gaimardi*	*Cyphastrea serailia*	
			*M. trabeculata*	*Galaxea fascicularis*	
			*M. hispida*	*Symphyllia agaricia*	
		450 m	Lower Zone- - - - - -	- - - - - - - - - - - - - - - - - - - -2 m- - - - - - - - - - - - - 450 m	
Seaward slope		Lower Zone	*Acropora formosa*	*Montipora foliosa*	*Acropora corymbosa*
			*A. affinis*	*Montipora solanderi*	*Acropora surculosa*
			*A. pacifica*	*Astreopora myriophthalma*	*A. affinis*
			*A. brueggemanni uncinata*	*Pavona decussata*	*A. brueggemanni uncinata*
			*A. humilis*	*Pavona lata*	*A. fizardi*
			*A. nasuta*	*Goniastrea retiformis*	*Acropora formosa*
			*Montipora trabeculata*	*Hydnophora microconos*	*Porites lutea*
			*M. hispida*	*Galaxea lamarckii*	*P. pukoensis*
			*Pavona frondiferi*	*G. aspera*	*Goniastrea yammanarii*
			*Porites lutea*	*Lobophyllia costata*	*Lobophyllia costata*
			*P. pukoensis*	*Lobophyllia corymbosa*	*Millepora latifolia*
			*Goniastrea pectinata*	*Merulina laxa*	*M. platyphylla*
			*Platygyra crosslandi*	*Merulina ampliata*	
			*P. rustica*	*Pectinia lactuca*	
			*Hydnophora contignatio*	*Turbinaria peltata*	*Simularia* sp.
			*Cyphastrea serailia*	*Fungia fungites*	(Softcoral)
			*Galaxea fascicularis*	*Fungia echinata*	
			*Symphyllia agaricia*	*F.* sp.	
			*Millepora platyphylla*	*Millepora latifolia*	
			*M. murrayi*	*Millipora intricata*	
					−6.6 m

In 1976 and 1977, a group of marine biologists led by Academician C. K. Tseng [46] studied the reef structure and zonation of the coral reef and algal ridge at Jinyindao atoll, Xisha Island. This baseline is valuable in documenting changes in the structure of the atoll. Based on the dominant coral species inhabiting the northeast and southwest transect of Jinyindao atoll at low summer tides, several zones were established. In the northeast (960 m), these zones are:

1. *Porites lutea* Zone (310 m),
2. *Heliopora coerulea* Zone (350 m),
3. *Acropora formosa* Zone (100 m),
4. Dead Coral Fragments Zone (80 m), and
5. Coral Algal Zone (120 m).

In the southwest (450 m), the zones are:

1. Coral Sand Zone (150 m),
2. *Heliopora coerulea* Zone (200 m),
3. *Acropora Zone* (70 m), and
4. Coral Algal Zone (30 m).

The records are valuable for references to monitor and assess the change of reef structure of the Xisha atolls.

#### (4) Conservation of mangrove forests

There are 37 mangrove and semi-mangrove species recorded from the China seas. Exploitation and construction in coastal zones and increased impacts caused by human activities in the coastal area have seriously damaged mangrove forests, which are exploited by local peoples for fuel and to obtain dyes. The total area of China's mangrove swamp has decreased since 1950, although some recovery has been observed since the 1990s (Table 10) [47], [48].

**Table 10:** Area of mangrove forests and number of mangrove species in China [51], [52].

Region	Mangrove area (ha)			Number of species		
	1950s	1990s	2001	True mangrove	Semi-mangrove	Total
Hainan	9,992	4,836		24	11	35
Guangxi	10,000	5,654		9	5	14
Guangdong	21,289	3,813		10	8	18
Hong Kong	85	9		11		
Aomen Macao	1	4		5		
Fujian	720	360		7	2	9
Taiwan	120	9		17		
Zhejiang	8	1		1		
**Total**	**42,000**	**14,877**	**22,024**	**26**	**11**	**37**

In addition to conservation measures to protect mangrove ecosystems from impacts caused by human activities, ecological and other basic studies of mangrove ecosystems and biodiversity of mangrove birds and insects are now being conducted with the support of the National Science Foundation and local governments. Useful information and valuable data related to biodiversity conservation have been collected. Recognizing the important role that mangroves play in protecting the shoreline against erosion, people have introduced and transplanted mangrove trees to conserve the swamp environment. Mangroves have also been introduced from neighboring countries.

#### (5) Seagrass field study and conservation

Species composition of seagrass flora is different in northern and southern China seas (Table 11) [43]. Temperate species belonging to the genus *Zostera* are abundantly distributed in the Yellow and Bohai seas, where six species have been recorded, including *Z. japonica*, a subtropical species commonly found much farther south in Hong Kong. *Phyllospadix japonica* and *P. iwatensis* are other temperate species commonly found in northern China.

**Table 11:** Seagrass species, distribution, and cover in southern China [47].

Province	Locality	Seagrass bed area (ha)	Main species
Guangdong	Liusha Bay	900	*Halophila ovalis, Halolule uninervis*
	Donghai Dao	9	*Halophila beccarii*
	Hailing Dao	1	*Halophila ovalis*
Guangxi	Hepu	540	*Halophila ovalis*, *Halolule uninervis, Zostera japonica, Halophila beccarii*
	Zhezhugang	150	*Zostera japonica, Halophila beccarii*
Hainan	Lian Gang	320	*Enhalus acoroides*, *Thalassia hemprichii*, *Cymodocea rotundata*, *Halophila ovalis*, *Halolule uninervis*
	Xincun Gang	200	*Enhalus acoroides*, *Thalassia hemprichii, Cymodocea rotundata, Halolule uninervis*
	Longwan SGB	350	*Enhalus acoroides*, *Thalassia hemprichii*, *Halophila ovalis*
	Sanyawan	1	*Enhalus acoroides*,*Thalassia hemprichii*
Hong Kong	ShenZhenWan	—	*Zostera japonica, Halophila ovalis*
	DapengWan	—	*Halophila ovalis*, *Ruppia maritime*

**Figure 9:**
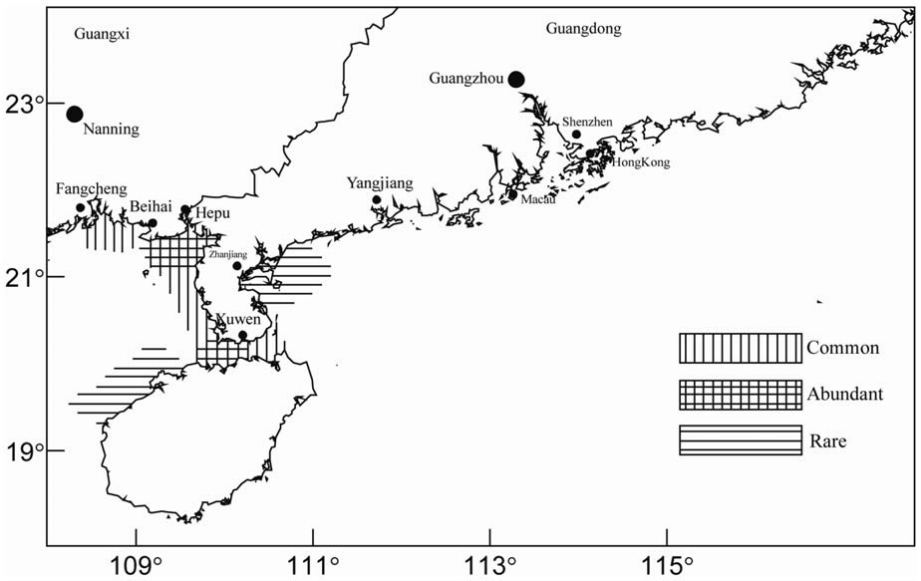
Distribution of *Dugong dugon* and seagrass fields along southern China coast.

In the absence of conservation practices, a serious decrease has occurred in the *Zostera* bed area along the coast of north China. However, seagrass fields in southern China are better protected than those in the north. In Hainan, a natural reserve has been established to protect seagrass beds of tropical species of the genera *Thalassia, Enhalus*, and *Cymodocea* [43]. The *Dugong dugon* is the most important seagrass grazer in southern China and is also an emblematic protected species (Figure 9). This species used to be abundant in the southern provinces of Guangxi, Guangdong, and Hainan with an estimated population of 216 individuals between 1958 and 1962. At present, despite its protected status, the dugong is rarely found and the species is considered to be endangered and almost collapsed.

#### (6) Stock enhancement of Chinese shrimp through juvenile release

**Figure 10:**
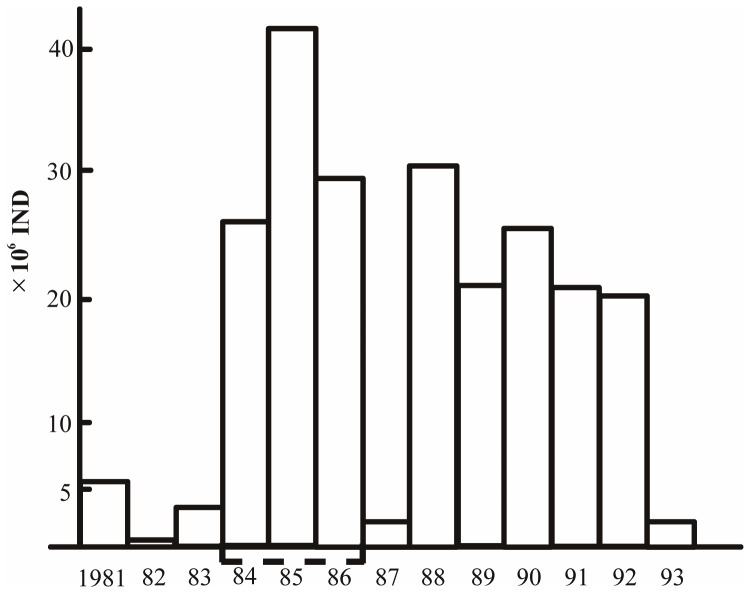
Estimated total number of individual Chinese shrimp *Fenneropenaeus chinensis*, Jiaozhou Bay in August (1981–1993).

To maintain sustainable development of natural stocks of shrimp, artificially cultivated juveniles of the Chinese shrimp *Fenneropenaeus chinensis* were released into Jiaozhou Bay, Yellow Sea, after a three-year monthly survey of oceanographic environments (physical and biological) and of the living resources in 1980–1982. After their release, a monthly monitoring survey was carried out in 1983. The same experiment was carried out again in 1984 and in subsequent years up to 1993 (Figure 10). The more than tenfold increase in the stock size [49] and the resulting high shrimp recapture in and out of the bay indicate the success of the experiment. A low catch in 1987 was due to a lack of juvenile shrimps available for release. A similar experience was recorded with the olive flounder *Paralichthys olivaceus*. The successful outcome of these experiments indicates that enhancement of marine living resources should be encouraged for sustainable development. Based on the experiences obtained in the experimental practices, large-scale release of Chinese shrimp fry was carried out from 1987 to 1992 along the Bohai Sea and the Yellow Sea coasts, and more than 5,000 tons of shrimp were recaptured.

### Progress in marine biodiversity study

Since the establishment of the Chinese Academy of Sciences (CAS), scientists in the Marine Organism Taxonomy and Phylogeny Study Group, Institute of Oceanology, have carried out taxonomy, ecology, and bioresources studies. Extensive collections of marine planktonic, benthic, and nektonic organisms have been made in coastal zones and shelf waters of the China seas, resulting in about 600,000 specimens of micro- and macroalgae, protozoans, invertebrates, and fishes deposited in the Marine Biological Museums, Chinese Academy of Sciences, in Qingdao and Guangzhou for taxonomic and systematic studies. Over a thousand papers and volumes of monographs have been contributed, of which the 33 volumes of *Fauna Sinica—Invertebrata*, and 7 volumes of *Flora Algarum Marinarum Sinicarum (Marine Algal Flora of China)* were published recently. The main taxonomic monographs are listed in Table 12.

**Table 12:** Main publications on marine fauna and flora of China's seas.

Name of publications		Volumes published
*Fauna Sinica*	Invertebrata	47 volumes by Science Press (25 volumes marine)
	Vertebrata	7+1 volumes
*Flora Algarum Marinarum Sinicarum*		8 volumes
*Studia Marina Sinica*		>22 volumes, Institute of Oceanology, Chinese Academy of Sciences (IOCAS)
*Studia Marina Sinca—Xisha Islands marine biota*		6 volumes
*CAS Nansha Islands Comprehensive Expedition, Science Reports*		South China Sea Institute of Oceanology (SCSIOCAS) >15 volumes
*Marine Species and Their Distribution in China's Seas*		HUANG Zongguo, editor, 1994. 20,278 species recorded (including fossil spp.) 2^nd^ ed. 2008
*Checklist of Marine Biota of China Seas*		LIU Ruiyu, editor, 2008. 22,629 species recorded.

A total of 1,577 new species, 87 new genera, 6 new families, and 1 new subclass have been discovered and described, and many more new records of distribution of marine species in China seas have been found. Results of studies on biodiversity and biogeography in China seas obtained before 1994 were integrated in *Marine Species and Their Distribution in China's Seas* [50] and summarized in the *Great National Physical Geography Atlas of People's Republic of China* - Oceanography Chart Set by Liu and Weng (in Liao [51]). They represent a significant advance in taxonomic and biodiversity studies on marine life. Further efforts have been made and advances achieved in the last 14 years. Many new taxa including species, genus, and family categories were found and described, leading to new understanding of the general features and characteristics of biotic components, biogeography, and biodiversity.

New findings have also been made since 2000 in the assessment of endangered species in China. The newly published *China Species Red List*, Vol. 1, 2A, 2B, and 3 [52]–[54] shows that the number of endangered marine invertebrate species (assessed by scientists of my research group adopting the new criteria of the International Union for Conservation of Nature, IUCN) have been distinctly increased. A new volume of biodiversity monograph entitled *Checklist of Marine Biota of China Seas* [28], published in 2008, integrates the above-mentioned new information and data. The Checklist systematically records the scientific names (in Latin and Chinese) of all species (totaling 22,629) of Chinese marine life in 46 major phyla, including original designation, the main synonyms, and the geographical distribution in China seas and the world oceans. The reference literature and systematic index of all taxa are also provided. A total of 5,118 more species (29.2%) were added to those (17,511 species) recorded in 1994. This major compilation indicates that great success and progress in taxonomy and biodiversity studies have been achieved in this short period of 14 years. The increase in number and percentage of marine species was particularly high in the dominant groups of invertebrates. The number of species of crustaceans, for example, increased by 50% to 4,320, and the number of mollusks increased by 53.1% to 3,914. The next is fish (3,012 species), Cnidaria (1,402 species), Polychaeta (1,067 species), Foraminifera (1,478 species), and Diatomeae (1,427 species). Table 2, based on the *Checklist of Marine Biota of Chins Seas*, shows the estimated number of undescribed species for all taxonomic groups. Because of inadequate sampling of certain groups in various habitats such as the deep sea and distant sea areas, the number of known species is quite low even in shallow coastal areas. Therefore the potential for discovery of new species in China is very high.

Shallow shelf water of the China seas is rich in living resources with high productivity, which strongly supports the marine fishery production. The annual production of both, fisheries (Table 13) and mariculture (Table 14) topped the world countries since the end of last millennium.

**Table 13:** Annual production of marine fisheries (catch) of China, 2007.

Production category	Subcategory	Annual production (tons)
**Sea area**	Bohai Sea	994,587
	Yellow Sea	2,887,797
	East China Sea	4,183,807
	South China Sea	3,210,494
	Others (Open sea)	1,158,695
	China total	12,435,480
**Fishery type**	Trawling	6,052,907
	Purse seine	705,101
	Gill net	2,314,878
	(Fixing) Pole net	1,788,887
	Longline	722,308
	Others	851,399
**Crustacea** (Shrimp)	*Acetes* spp.	608,096
	*Penaeus* spp.	111,299
	*Trachysalambria* spp.	344,978
	*Oratosquilla* spp.	306,969
	Total shrimp	1,513,366
**Crustacea** (Crab)	*Portunus* spp.	350,631
	*Scylla* spp.	68,588
	*Charybdis* spp.	64,829
	Total crab	557,036
**Mollusca** (Cephalopoda)	*Sepiella* spp. Cuttlefish	152,754
	Squid	718,200
	Octopus	139,416
	Total Cephalopoda	1,047,713
**Mollusca** (Shellfish: bivalves and snails)		743,617
**Others**	Jellyfish	223,868
	Total others	316,670
**Seaweeds**		32,847

**Table 14:** Mariculture production of China, 2007.

Species group	Tonnage	Area
Fishes	688,563 t	60,733 ha
Crustaceans	919,008 t	279,648 ha
Mollusca (shellfish)	9,938,377 t	791,938 ha
Seaweed	1,355,536 t	77,922 ha
Others	171,916 t	121,237 ha
**Total mariculture production**	**13,073,400 t**	**1,331,478 ha**

The Marine Organism Taxonomy and Phylogeny Lab at IOCAS has assessed species richness of marine life per 100 km coastline (Table 5). The results revealed that species richness depends upon the intensity of the exploration surveys. For example, 501 species of macrobenthic species were recorded from Quanzhou Bay by Huang [36], while about 770 species were reported in a more recent monitor survey carried out by the Marine Environment and Fishery Resources Monitor Center of Fujian Province [35] from the same Bay (Table 15). These results indicate that intensive exploration and collection should be made to obtain accurate data of species richness.

**Table 15:** Number of species recorded from Quanzhou Bay.

Groups	Number of species, 2004	Number of species, 2009
Phytoplankton	104	197
Zooplankton	82	166
Shallow-water macrobenthos	169	152
Intertidal macrobenthos		225
Fish eggs, larvae, and juveniles	—	21
Nekton	142	83
Fouling organisms	101	
Total	501	770

**Figure 11:**
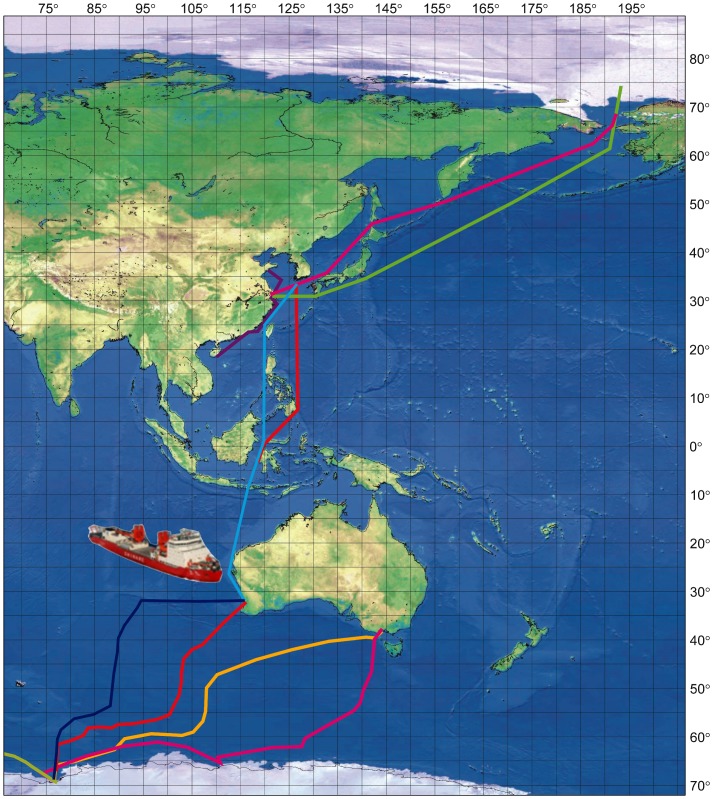
Marine biodiversity studies carried out by the Census of Marine Zooplankton Project, including the “Arctic Ocean, Equator, Antarctic Ocean” trans-equator transect.

The IOCAS study group led by Sun has participated in the world biodiversity project Census of Marine Life (CoML) since 2004. In the Census of Marine Zooplankton Project, more than 1,000 samples have been collected, including 300 samples from the “Arctic Ocean, Equator, Antarctic Ocean” trans-equator transect (Figure 11). Regular zooplankton sampling is carried out seasonally in the Yellow Sea and the East China Sea. In DNA barcoding studies, data of 148 species of zooplankton and 60 benthic shrimps have been submitted.

## Discussion

### Threats to marine biodiversity and conservation efforts

With the rapid development of industrial production in coastal cities, an increase in the intensity of fishing activity in the inner shelf area and a corresponding rise in environmental pollution and decline of living resources, the high biodiversity and richness of marine species and living resources in China seas has seriously decreased. Sustainable development of fishery production seems to be difficult. China has fully exploited the living resources of its coasts and continental shelf. Indeed, many species of living resources of China's coastal areas are overexploited. Examples are the large yellow croaker *Larimichthys crocea* [15], the Chinese shrimp *Fenneropenaeus chinensis* [55], and the cuttlefish *Sepiella japonicus* [56], for which captures in recent years decreased significantly due to population decline and collapse [15], [20].

Tables 13 and 14 show annual fishery production in 2007. The marine catch production of 12,435,480 tons and mariculture production of 13,073,400 tons were valued at ¥104,510.79 million ( = $16.6 billion) and ¥110,796.60 million ( = $17.6 billion), respectively, for a total of ¥ 215,307.39 million ($34.2 billion). Overexploitation is therefore the most serious problem facing marine biodiversity because it prevents not only recruitment and recovery of the stocks but also the possibility of sustainable development.

Besides the overexploitation of fishery resources, the major threat to the biodiversity of the China seas is environmental deterioration (pollution, coastal construction), particularly in the brackish waters of estuarine environments, which are characterized by high productivity and represent spawning and nursery areas for several species. In the long term, climate change is also a major threat. To maintain and conserve the highly diversified marine biota and the rich living resources of the China seas, the government of China has adopted laws and regulations for their conservation and at the same time has established many natural conservation areas (reserves) and areas or periods of time in which fishing is forbidden. Various research projects have been approved and financially supported. Changes in the biodiversity of various habitats such as intertidal mud flats, coral reefs, and mangrove swamps have been monitored and studied. An assessment of endangered species of major vertebrate groups (mammals [57], amphibians and reptiles [58], birds [59], and fishes [60]) was published in *China Red Data Book of Endangered Animals* in the 1990s. More recently, a new Red List of plant and animal species (terrestrial, freshwater, and marine) has been published, based on historical as well as new data, with the threatened category of species assessed using new IUCN criteria [61]. Since 2004 *The China Species Red List* has documented the increasing number of species endangered by the impact of human activities (mainly overexploitation and environmental pollution) and by global climate change [52]–[54]. *The China Species Red List* is not encouraging as many marine species are regarded as “endangered” because of overexploitation for the seafood market or for “fine art” collections. Following are a few examples of this trend:

- The Chinese shrimp *Fenneropenaeus chinensis* is a large endemic species with a high economic value and a high production of more than 40,000 tons in 1979 in north China (mainly Yellow Sea and Bohai Sea). The current population of this shrimp is very small in its natural habitat in North China waters because of overfishing and deterioration of spawning and nursing grounds in the Bohai and Yellow seas. Lost of population in the South China Sea since the end of the last century, the species was assessed as endangered in 2005 [53].
- The large yellow croaker *Larimichthys crocea*, another of the most important fisheries resources, has been seriously overfished. Only 167 young fishes were caught during the 1997–2000 exploration survey, leading scientists to estimate that the population of this species on the East China Sea Shelf is 71 tons. Considering that the highest catch of this species was 180,000 tons in the 1980s, the large yellow croaker has also been assessed as endangered [15], [54].
- The horseshoe crab *Tachypleus tridentatus* historically has been abundant in the South China Sea, particularly in the Gulf of Tonkin, but is now considered endangered because of overexploitation [45].
- Among the 256 species of scleractinian corals, 26 species have been assessed as endangered while all the others are considered vulnerable endangered.
- Among the Mollusca, 23 species are considered endangered.
- Among the Crustacea Decapoda, 56 species are now endangered. One of these species is the Chinese spiny lobster *Panulirus stimpsoni*, which shows a population collapse due to overexploitation. Although the population of this species has been large in rocky shores in western Guangdong, since the 1990s, only very few young individuals have been collected.
- Among the fish, 270 species are endangered. Of these, 19 species are critically endangered and 4 are extinct [62].
- Among the 150 species of holothurid echinoderms, 53 are considered endangered due to serious overexploitation.

To address current problems in marine biodiversity studies, the following suggestions have been put forward [8]:

- Strengthen the collection of materials (specimens) for marine biodiversity study by carrying out a biodiversity background value survey and deep-sea collection cruises to discover new species and reveal the past and present abundance of major species and biological communities while forecasting their future. Intensive collections and study of deepwater marine biodiversity should be made to discover new species and to reveal past, present, and future trends of major species and biological communities.
- Carry out biodiversity monitoring surveys in various habitats around the country to assess their present status and to understand the processes and mechanisms of global climate change and human activities that have an impact on biodiversity.
- Strengthen basic research on change of marine biodiversity, particularly the assessment and conservation of biodiversity and endangered species for sustainable development.
- Minimize the disparity between the study and conservation of marine and terrestrial (including freshwater) biodiversity, and effective management. To achieve this, young scientists, particularly taxonomists, should be trained to study different biotic groups systematically.
- Strengthen conservation management to achieve more effective management of fisheries resources.

### Known, unknown, and unknowable

Although significant advances have been made in marine biodiversity research in China since the 1950s through data collection and the formal description of species, much remains to be done. The biogeographic features and habitat of the deep sea and the southern China seas have not been well explored. Taxonomic coverage of many groups such as those that comprise the meio- and microbenthos—the nematodes, harpacticoids, and ostracod crustaceans, etc.—is incomplete in our present data, which comes largely only from northern Chinese waters. The study of ecosystem structure and function has been particularly neglected in “the utmost environment”—the Yellow Sea Cold Water Mass—which is considered a refuge for North Pacific Temperate Fauna. Exploration and study of the bathyal and abyssal depths of the South China Sea, including the sea mountains, the hydrothermal vents, and cold-water seeps as well as the abyssal plain and rocky trenches, should be strengthened.

## Supporting Information

Table S1 **Principal institutions for marine science research in China.**
(DOC)

Table S2 **Scientists working on biodiversity, taxonomy, and systematics of marine biota in China.**
(DOC)
