# Supplementary material for: Status of Marine Biodiversity of the China Seas
Source: PLoS One. 2013 Jan 8;8(1):e50719. doi: 10.1371/journal.pone.0050719 (PMC3540058; doi:10.1371/journal.pone.0050719)
Supplement: Table S1 — Principal institutions for marine science research in China. (DOC) [file pone.0050719.s001.doc]

Table S1. Principal institutions for marine science research in China.

| **Institution** | | **Location** | **General introduction, National laboratories, Key laboratories** |
| --- | --- | --- | --- |
| Chinese Academy of Sciences | Institute of Oceanology | Qingdao | [Experimental Marine Biological Laboratory, CAS](http://english.qdio.cas.cn/rh/rd/embl/) |
|  |  |  | [Key Laboratory of Marine Ecology and Environmental Sciences, CAS](http://english.qdio.cas.cn/rh/rd/klmees/) |
|  |  |  | [Key Laboratory of Ocean Circulation and Wave Studies, CAS](http://english.qdio.cas.cn/rh/rd/klocws/) |
|  |  |  | [Key Laboratory of Marine Geology and Environment, CAS](http://english.qdio.cas.cn/rh/rd/klmge/) |
|  |  |  | [R&D Center of Marine Biotechnology, CAS](http://english.qdio.cas.cn/rh/rd/cmb/) |
|  |  |  | [R&D Center of Marine Environmental Engineering and Technology](http://english.qdio.cas.cn/rh/rd/cmee/) |
|  |  |  | [Department of Marine Organism Taxonomy and Phylogeny](http://english.qdio.cas.cn/rh/rd/motp/) |
|  |  |  | [Center for Marine Corrosion and Protection](http://english.qdio.cas.cn/rh/rd/cmcp/) |
|  | South China Sea Institute of Oceanology | Guangzhou | State Key Laboratory of Tropical Oceanography, SCSIO, CAS |
|  |  |  | Key Laboratory of Marine Bioresources Sustainable Utilization (LMB) |
|  |  |  | Key Laboratory of Tropical Marine Environmental Dynamics (LED), CAS |
|  |  |  | Guangdong Key Laboratory of Applied Marine Biology (LAMB) |
|  |  |  | Guangdong Key Laboratory of Marine Materia (LMM) |
|  |  |  | Key Laboratory of Marginal Sea Geology, SCSIO, CAS |
|  | Institute of Zoology | Beijing | Fish and crustacean taxonomy |
|  |  |  | State Key Laboratory of Reproductive Biology |
|  |  |  | Key Laboratory of Animal Ecology and Conservation Biology, CAS |
|  |  |  | Key Laboratory of Zoological Systematics and Evolution, CAS |
|  | Institute of Coastal Zone Research | Yantai | Crustacea and decapoda taxonomy; Coastal zone ecosystem and living resources; Ichthyology and protozoology; Freshwater ecosystem and living resources, Aquaculture |
|  |  |  | Environmental Microanalysis & Monitoring (EMM) Laboratory |
|  |  |  | Coastal Information Integration and Application Laboratory |
|  |  |  | Environmental Analytical Chemistry Laboratory |
|  |  |  | Coastal Ecology and Environment Laboratory |
|  |  |  | The Laboratory of Coastal Wetland Ecology |
|  |  |  | Pollution Process and Control Laboratory |
|  |  |  | Biological resources Laboratory |
|  | Institute of Hydrobiology | Wuhan | Center for Aquatic Biodiversity and Resource Conservation |
|  |  |  | Center for Freshwater Ecology |
|  |  |  | Center for Fish Biology and Fishery Biotechnology |
|  |  |  | Center for Water Environment Engineering |
|  |  |  | Center for Water Environment and Human Health |
|  |  |  | Center for Algal Biology and Applied Research |
| Ministry of Education | Xiamen University | Xiamen | School of Life Sciences, College of Oceanography and Environmental Science |
|  |  |  | Department of Oceanography |
|  |  |  | Department of Environmental Science and Engineering |
|  |  |  | Department of Marine Technology and Engineering |
|  | Ocean University of China | Qingdao | National Laboratory for Marine Science and Technology (NaLMaST) |
|  |  |  | Key Laboratory of Physical Oceanography, MOE |
|  |  |  | Key Laboratory of Ocean Remote Sensing, MOE |
|  |  |  | Key Laboratory of Mariculture, MOE |
|  |  |  | Key Laboratory of Marine Drugs, MOE |
|  |  |  | Key Laboratory of Marine Ecological Environment, MOE |
|  |  |  | Key Laboratory of Marine Chemistry Theory and Technology, MOE |
|  |  |  | Key Laboratory of Submarine Geosciences and Prospecting Techniques, MOE |
|  | Shanghai Ocean University | Shanghai | Key Laboratory of Aquatic Genetic Resources and Aquacultural Ecology, MOA |
|  |  |  | College of Marine Science |
|  |  |  | College of Fisheries and Life Science (Fishery resources, Fishery engineering science) |
|  | Tongji University | Shanghai | National Laboratory of Marine Geology |
|  |  |  | The State Key Laboratory of Marine Geology at Tongji University conducts research on environmental evolution and natural resources in oceans and neighboring continental regions. Programs emphasize deep-sea basic research through international collaboration. |
|  | University of Hong Kong | Hong Kong | The Swire Institute of Marine Science, Croucher Laboratory of Environmental Hydraulics |
|  | Chinese University of Hong Kong | Hong Kong | The Yuen Yuen Research Centre for Satellite Remote Sensing, Simon F.S. Li Marine Science Laboratory |
|  |  |  | Department of Biology (Major research areas: Plant and Fungal Biotechnology, Marine Biotechnology, Microbiology/Microbial Biotechnology, Traditional Chinese Medicine (TCM) / Natural Products / Health, Sytematics, Physiology, Marine Biology, Environmental Biology / Ecology, Food Sciences) |
|  | Nanjing Normal University | Nanjing | Sea mammal taxonomy and conservation |
|  |  |  | College of Life Sciences /Lab / Biotechnology |
|  | Zhongshan University | Guangzhou | National Engineering Research Center for Marine Biological Technology of South China Sea |
|  |  |  | Research Institute of Economic Aquatic Animals |
|  |  |  | Marine Biotechnology Center of South China Sea |
|  | Jinan University | Guangzhou | Microalgae and phytoplankton |
|  |  |  | Research Center of Hydrobiology |
|  |  |  | Department of Ecology |
|  | Liaocheng University | Liaocheng Shandong | College of Life Sciences, (Laboratory of Nemotodes) Microbenthos Ecology |
| State Oceanic Administration | First Institute of Oceanography | Qingdao | Distributions and variabilities of natural environmental elements in Chinese seas, their adjacent oceans and polar seas, marine resources and environmental geology, the generating mechanism and prediction method of marine disasters, variabilities of marine ecology environment, marine high technology development, and marine comprehensive management sciences. |
|  |  |  | Key Lab of Marine Science and Numerical Modeling |
|  |  |  | Key Lab of Marine Resource and Environmental Geology |
|  |  |  | Key Lab of Science and Engineering for Marine Ecology and Environment |
|  |  |  | Key Lab of Marine Biological Active Substances |
|  |  |  | Key Lab of Marine Remote Sensing Science and Marine Dynamic Information System Technology |
|  |  |  | Research Center for Coastal Zone and Island Science and Engineering Survey |
|  |  |  | Research Center for Ocean Mapping and Engineering Information |
|  |  |  | Research Center for Marine Monitoring and Experimental Technology |
|  |  |  | Research Center for Marine Economy Planning and Management |
|  |  |  | Key Lab of Material Transport and Transformation in Environmental and Life Processes |
|  |  |  | Marine Information Center |
|  | Second Institute of Oceanography | Hangzhou | Seafloor geosciences and deep-sea survey technology |
|  |  |  | National Laboratory of Satellite Ocean Environment Dynamics |
|  |  |  | Key Laboratory of Seafloor Geosciences, SOA |
|  |  |  | Key Laboratory of Marine Ecosystem and Biogeochemistry, SOA |
|  | Third Institute of Oceanography | Xiamen | Marine biotechnology and resource development, ocean-atmosphere chemistry and global change, ocean ecosystem and environmental protection, application oceanography of Taiwan strait and tropical marginal sea. |
|  |  |  | Biodiversity Lab |
|  |  |  | Key Laboratory of Marine Biological Genetic Resources, SOA |
|  |  |  | Key Laboratory of Ocean-atmosphere Chemistry and Global Change, SOA |
|  | National Marine Environmental Monitoring Center | Dalian | Department of Marine Environmental Monitoring Quality Technology |
|  |  |  | Department of Sea Area Use Management Technology |
|  |  |  | Department of Marine Environmental Monitoring Remote Sensing |
|  |  |  | Department of Marine Environmental Monitoring Information |
|  |  |  | Department of Marine Environmental Chemistry |
|  |  |  | Department of Marine Environmental Dynamics |
|  |  |  | Department of Marine Environmental Ecology |
|  |  |  | Key Laboratory of Coastal Ecological Environment, SOA |
| Ministry of Agriculture | Yellow Sea Fisheries Research Institute | Qingdao | Key Laboratory of Sustainable Development of Marine Fisheries, Ministry of Agriculture |
|  |  |  | Laboratory of Stock Assessment and Ecosystem Management |
|  |  |  | Laboratory of Marine Fishery Environment and Bio-remediation |
|  |  |  | Laboratory of Mariculture Ecology and Carrying Capacity |
|  |  |  | Laboratory of Maricultural Organism Disease Control and Molecular Pathology |
|  |  |  | Laboratory of Genetic Resources and Breeding |
|  |  |  | Laboratory of Marine Products and Enzyme Engineering |
|  |  |  | Division of Quality Test and Safety Management of Aquatic Products |
|  |  |  | Division of Marine Fish Culture and Engineering |
|  |  |  | Division of Food Engineering and Nutrition Research |
|  | East China Sea Fisheries Research Institute | Shanghai | Management and evaluation of fishery resources, development of pelagic and polar fishery resources, monitoring and protection of the biological environment for fishery, fishery information technology, aquaculture carrying capacity and health culture, aquatic biotechnology, aquatic food safety and quality control, fishery resources and organism products, catching technology and fishery engineering, culture biology in special habitat, aquatic standardization. |
|  | South China Sea Fisheries Research Institute | Guangzhou | Protection and application of fishery resources, biological environments of fishery, healthy culture of aquatic product, biotechnology of genetic breeding, disease control of aquatic product, aquatic product processing and comprehensive utilization, safety and quality control of aquatic product, fishery equipment and engineering technology, fishery information. |
|  |  |  | Key Laboratory of Fishery Environments |
| Others | Beijing Natural History Museum | Beijing | Taxonomy and systematics of fishes and crustaceans |

Legend:

CAS: Chinese Academy of Sciences

MOE: Ministry of Education

SOA: State Oceanic Administration
