# Supplementary material for: Status of Marine Biodiversity of the China Seas
Source: PLoS One. 2013 Jan 8;8(1):e50719. doi: 10.1371/journal.pone.0050719 (PMC3540058; doi:10.1371/journal.pone.0050719)
Supplement: Table S2 — Scientists working on biodiversity, taxonomy, and systematics of marine biota in China. (DOC) [file pone.0050719.s002.doc]

**Table S2. Scientists working on biodiversity, taxonomy, and systematics of marine biota in China*.***

Superkingdom Prokaryota

## Kingdom Bacteria

Phyla Chloriobi, Proteobacteria, Firmicotes, Actinobacteria, Bacteroidetes

- Prof. Xiaomei ZHANG

Phylum Cyanobacteria: Profs. Bangmei XIA, Lanping DING

Superkingdom Eukaryota

Kingdom Chromista

Phylum Diatomeae: Prof. Yahui GAO and group,

Phyla Chrysophyta, Xanthophyta, Cryptophyta, Prymnesiophyta: Yahui GAO and Group

Phylum Phaeophyta: Prof. Bangmei XIA

Kingdom Plantae

Phylum Rhodophyta: Bangmei XIA

Phylum Chlorophyta: Bangmei XIA

Phylum Pteridophyta: Lanping DING, PhD

Phylum Magnoliophyta (Angiospermae): L. DING

Kingdom Fungii: Assoc Prof. Li TIAN

Phyla Zygomycota, Ascomycota, Basidiomycota, Deuteromycota

Kingdom Protozoa

Phylum Granuloreticulosa Class Foraminifera: Academician Shouyi ZHENG,

- & Senior Engineer Zhaoxian FU
- Academician Pinxian WANG

Phylum Dinozoa: Prof. Douding LU

Phylum Ciliophora: Prof. *Wei-bo SONG,

Phylum Radiozoa: Prof. Zhiyuan TAN,

- Prof. Muhong CHEN

Phylum Percolozoa Kuidong XU

Kingdom Animalia

Phylum Porífera: Assoc. Prof. Jinhe LI

Phylum Cnidaria: Prof. Zhican TANG

- Medusozoa Prof. Shangwu GAO
- Medusozoa Prof. Huixin HONG
- Medusozoa Prof. Zhenzu XU

Phylum Ctenophora: Prof. Qingchao CHEN

Phylum Myxozoa: Prof. Kuidong XU

Phylum Platyhelminthes, Class Trematoda: Prof. Jiuwei SHEN

Phylum Entoprocta: Xixing LIU

Phylum Nematoda: Prof. Zhi-nan ZHANG

- Prof. Yong HUANG

Phylum Nemertea: Prof. Shi-chun SUN

Phylum Phoronida: Prof. Xixing LIU

Phylum Bryozoa: Prof. Xixing LIU, Huilian LIU

Phylum Brachiopoda: Prof. Xixing LIU

Phylum Sipuncula: Prof. Zgucan TANG

Phylum Echiura: Prof. Xixing LIU

Phylum Annelida, Class Polychaeta: Prof. Ruiping SUN, Yanli LEI

- Prof. Dejian YANG
- Prof. Qi-quan WU

Phylum Mollusca

- Prof. Qi-qian ZHUANG Bivalvia
- Prof. Jinxiang JIANG
- Prof. Yingya CAI

Phylum Arthropoda, Subphylum Crustacea

- Academician Ruiyu LIU (JYLiu) Cirripedia, Nebaliacea, Stomatopoda
- Mysidacea, Decapoda Dendrobranchia, Alpheidoidea, Reptantia; Xiphosura
- Prof. Qingchao CHEN & Group
- Cladocera, Copepoda, Ostracoda, Amphipoda, Hyperiidea, Euphausiacea,
- Prof. Xinzheng LI Decapoda Caridea, Stenopidea.
- Prof. Xianqiu REN Cirripedia, Amphipoda Gammaridea, Caprellidea
- Prof. Yongliang WANG Stomatopoda, Decapoda Anomura
- Associate Prof. Zhongli SHA Caridea Alpheoidea
- Dr. Wei JIANG Decapoda Brachyura Pinnotheroidea, Goneplacoidea
- Dr. Wenliang LIU Decapoda Nephropsidea, Thalassinoidea
- Prof. Ruixiang CHEN: Ostracoda, Hyperiidea)
- Prof. Si-liang YANG Decapoda, Stomatopoda

Phylum Chaetognatha: Prof. Yichang XIAO

Phylum Echinodermata: Prof. YulinLIAO

Phylum Hemichordata: Prof. Fengshan XU

Phylum Urochordata (Tunicata): Prof. Qingchao CHEN Appendiculata

- Prof. Xiuming HUANG

Phylum Chordata:

†Cephalochordata: Prof. FengshanＸU Amphioxi

†Vertebrata: Pisces

- Prof. Jiakun SONG
- Prof. Hanlin WU
- Prof. Si-zhong LI
- Prof. Pei-chi YUE
- Prof. Yunfei WU
- Prof. Jing LIU
- Prof. KuangChao SHAO Group

Amphibia and reptiliformes: Academician Ermi ZHAO

Aves: Associate Prof. Zhihua MA

Sea mammals: Prof. Kaiya ZHOU
